# Effects of water-nitrogen interaction on photosynthetic-stoichiometric balance in potato: a leaf physiological and ecological perspective

**DOI:** 10.3389/fpls.2025.1729301

**Published:** 2025-12-10

**Authors:** Bin Du, Juan Yin, Yingpan Yang, Jinze Li, Ying Tang

**Affiliations:** 1 School of Civil and Water Engineering, Ningxia University, Yinchuan, Ningxia, China; 2 Engineering Research Center for Efficient Utilization of Water Resources in Modern Agriculture in Arid Regions, Yinchuan, China; 3 Ningxia Research Center of Technology on Water-saving Irrigation and Water Resources Regulation, Yinchuan, China; 4 Hydraulic Research Institute of Ningxia Hui Autonomous Region, Yinchuan, Ningxia, China; 5 Field Scientific Observation and Research Station for Agricultural Irrigation in Ningxia Yellow River Diversion Irrigation Area, Ministry of Water Resources, Yinchuan, Ningxia, China; 6 Ningxia Dry Farming, Water Saving and Efficient Agricultural Engineering Technology Research Center, Yinchuan, Ningxia, China

**Keywords:** water-nitrogen interaction, potato, photosynthetic characteristics, plant ecological stoichiometry, random forest model

## Abstract

Potato, a globally important food crop, plays a key role in ensuring food security and poverty alleviation. Addressing the prominent contradiction between water scarcity and low nitrogen use efficiency in potato production in the arid regions of northwest China, this study conducted a water-nitrogen regulation experiment to explore the synergistic effects of water-nitrogen management on potato yield and physiological ecology. Irrigation treatments included the full irrigation quota (2250 m^3^ ha^-1^, W3), 20% water-saving (1800 m^3^ ha^-1^, W2), and 40% water-saving (1350 m^3^ ha^-1^, W1), while nitrogen application treatments consisted of the full nitrogen rate (195 kg ha^-1^, N3), 20% nitrogen reduction (156 kg ha^-1^, N2), 40% nitrogen reduction (117 kg ha^-1^, N1), with the local conventional practice as the control (CK). The results showed that the W2N2 treatment had the highest chlorophyll content, which was significantly higher than other treatments and 2.87% higher than CK on average. Under water-nitrogen interaction, potato leaf gas exchange parameters first increased and then decreased with growth stages, peaking at the tuber formation stage, and the W2N2 treatment was significantly superior to other combinations. For nutrient contents in various plant organs, the W2N2 treatment also achieved the highest total nitrogen and total phosphorus contents, which were significantly higher than other treatments. The distribution ratios of nutrient contents in different organs varied with water-nitrogen treatments across years. Based on quadratic regression analysis and the TOPSIS model, the optimal water-nitrogen coupling pattern for potato cultivation in the arid zone of central Ningxia was determined as W2N2, providing theoretical and technical references for efficient potato cultivation with water-saving and nitrogen-reducing practices in arid regions.

## 1 Introduction

Potato (*Solanum tuberosum* L.) is a globally important food crop that plays a vital role in ensuring food security and reducing poverty (Ma et al., 2021). However, its cultivation and yield formation are limited by multiple factors, with drought stress and nitrogen fertilizer management being the primary constraints on production (Fandika et al., 2016; Satognon et al., 2021). Practical experience has demonstrated that optimizing irrigation practices to improve crop water use efficiency can enable irrigated farmland to achieve high yields with minimal water input. This approach is not only a crucial strategy to alleviate water scarcity and mitigate ecological and environmental issues in arid and semi-arid regions but also a key research focus within water-saving agriculture (Kang et al., 2025). Concurrently, the rapid increase in potato yield has historically depended on high inputs of chemical nitrogen fertilizers; however, prolonged excessive and improper fertilization has significantly reduced nitrogen use efficiency (Chen et al., 2024). Studies have shown that moderate reductions in nitrogen application can enhance nitrogen use efficiency, whereas excessive reductions result in yield decline (Lombardo et al., 2020). Therefore, achieving water-saving irrigation without compromising potato yield, alongside reasonable reductions in nitrogen fertilizer application, has become a critical scientific challenge in sustainable agricultural development.

Leaf stomatal exchange parameters, as core indicators of plant physiological ecology, directly reflect the gas exchange capacity between potato leaves and the external environment (Yun et al., 2020), and are closely linked to yield formation (Fu et al., 2021). The SPAD value, an important technical parameter for assessing chlorophyll concentration, is significantly affected by water stress, which in turn impacts potato chlorophyll content and tuber yield (Rolando et al., 2015). The coupling of water and nitrogen profoundly influences crop growth, yield, net photosynthetic rate, transpiration rate, and intercellular carbon dioxide concentration (Hong et al., 2022). Enhancing water and fertilizer use efficiency is essential for sustainable potato production (Kifle and Gebretsadikan, 2016; Chang et al., 2018), with studies showing that optimized irrigation combined with a 25% substitution of nitrogen fertilizer by manure can synergistically improve potato yield (Wang et al., 2024). Carbon, nitrogen, and phosphorus are the three fundamental elements driving ecosystem processes (Hao et al., 2017). Among these, carbon constitutes the structural basis of plants, while nitrogen and phosphorus are the primary nutrient elements limiting carbon assimilation, plant growth, and other biological functions (Sun et al., 2014; Gao et al., 2019). Nitrogen addition has been shown to significantly decrease the C:N ratio in plants and soils, increase plant N:P ratios, and exacerbate microbial carbon and phosphorus limitations, thereby advancing understanding of nutrient cycling processes in terrestrial ecosystems affected by global nitrogen deposition (Xu et al., 2022). Variations in plant nutrient stoichiometric ratios serve as sensitive indicators of changes in nutrient availability, which are reflected in plant physiological responses (Yan et al., 2015). Investigations into the ecological stoichiometric characteristics of plant carbon, nitrogen, and phosphorus help elucidate growth regulation mechanisms, survival strategies, and the effects of different plant organs and water-nitrogen interaction regimes on plant physiology (Chang et al., 2024). Han et al. (2011)pioneered a comprehensive analysis of nitrogen and phosphorus stoichiometry in the leaves of over 1900 plant species in China, revealing the relative stability of these limiting elements and their consistent responses to environmental fluctuations. Consequently, research on the effects of water-saving irrigation combined with nitrogen fertilizer reduction on photosynthesis, plant nutrient dynamics, and stoichiometry is both necessary and timely.

Although previous studies have examined the effects of water-nitrogen interactions on photosynthetic parameters, research on the dynamic changes in leaf gas exchange parameters, accumulation and distribution of carbon, nitrogen, and phosphorus, and their stoichiometric ratios throughout the entire growth period of potato under combined water-saving irrigation and nitrogen reduction management in the arid regions of Northwest China remains limited. Notably, a systematic investigation into the mechanisms by which water-nitrogen interactions influence yield through regulation of photosynthetic physiology and nutrient stoichiometric balance is still lacking. Addressing this gap, the present study employs field experiments to analyze the temporal dynamics of leaf gas exchange parameters; carbon, nitrogen, and phosphorus contents; nutrient accumulation and distribution ratios; stoichiometric ratios; and nitrogen and phosphorus limitation status across different growth stages of potato under varying water-nitrogen interaction regimes in the arid Northwest China region. The study aims to elucidate the interrelationships among leaf stomatal exchange characteristics, nutrient accumulation and distribution, stoichiometric balance across plant organs, and yield under water-nitrogen interaction, thereby providing a theoretical foundation and practical guidance for understanding the regulatory mechanisms of water-nitrogen interaction on potato photosynthetic physiology and stoichiometric equilibrium.

## 2 Materials and methods

### 2.1 Experimental site overview

**Figure 1:**
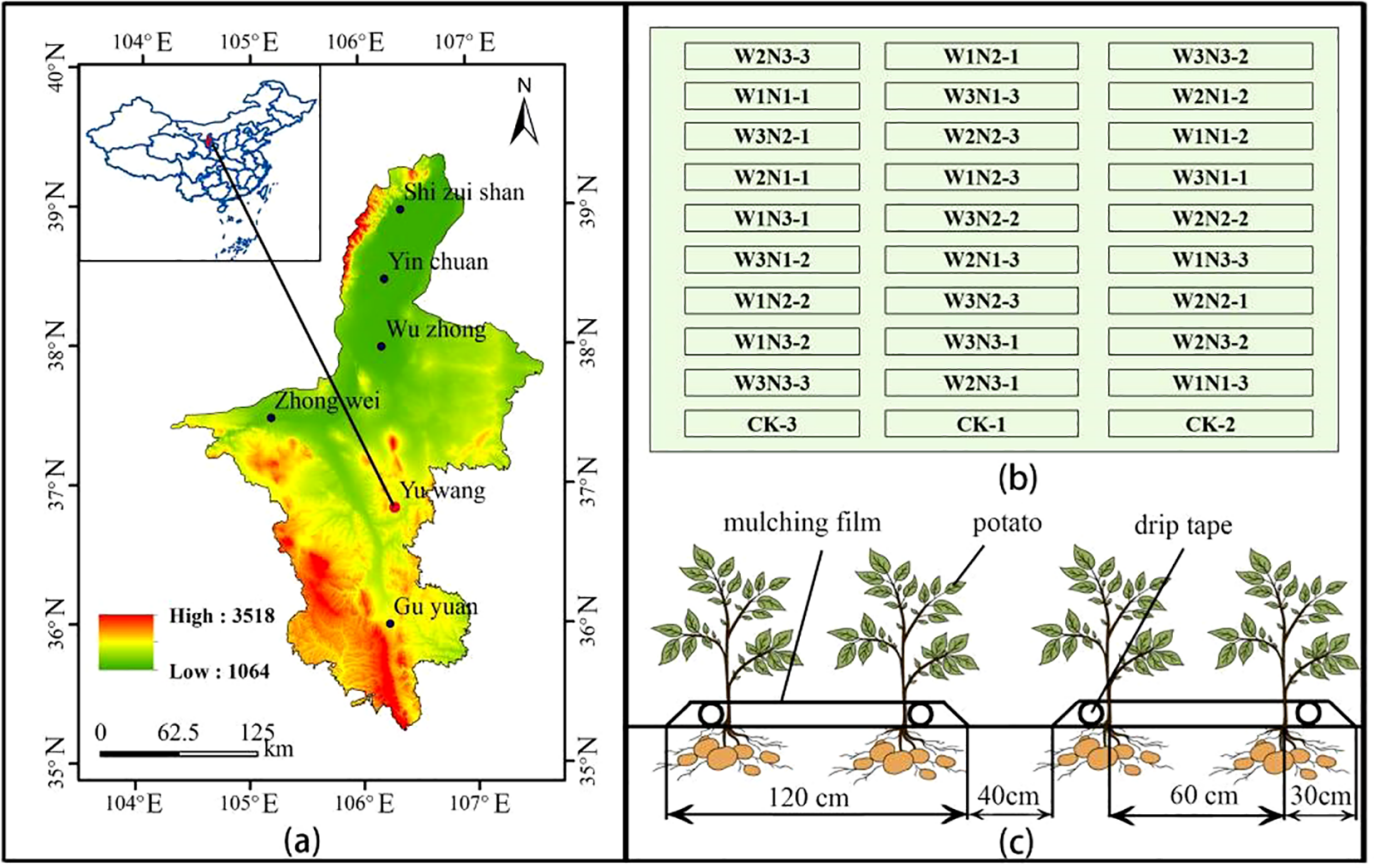
Overview of the experimental area. Panel **(a)** shows the location of the experimental area, Panel **(b)** illustrates the layout of the plot experiment, and Panel **(c)** presents the potato planting pattern.

The experiment was conducted during 2022–2023 in Yuwang Town (36°48′2″N, 106°21′53″E, altitude 1489 m), a typical area of the central arid zone in Ningxia, northwest China, as specifically shown in **Figure 1a**. The experimental area features a continental arid climate characterized by significant diurnal temperature variations, low precipitation, and intense evaporation. The multi-year average rainfall is approximately 270 mm, with annual evaporation reaching about 2,325 mm. The groundwater table lies below 10 m depth. The experimental soil is classified as sandy loam, with basic physical-chemical properties of the 0–40 cm tillage layer before sowing as follows: bulk density 1.41 g cm^-3^, field water-holding capacity 21.8%, pH 8.57, total salt content 0.6 g kg^-1^, organic matter content 6.65 g kg^-1^, alkaline hydrolysis nitrogen content 38 mg kg^-1^, available phosphorus content 3.94 mg kg^-1^, available potassium content 130 mg kg^-1^, total nitrogen content 0.27 g kg^-1^, total phosphorus content 0.64 g kg^-1^, and total potassium content 17.4 g kg^-1^.

### 2.2 Experimental design

**Figure 2:**
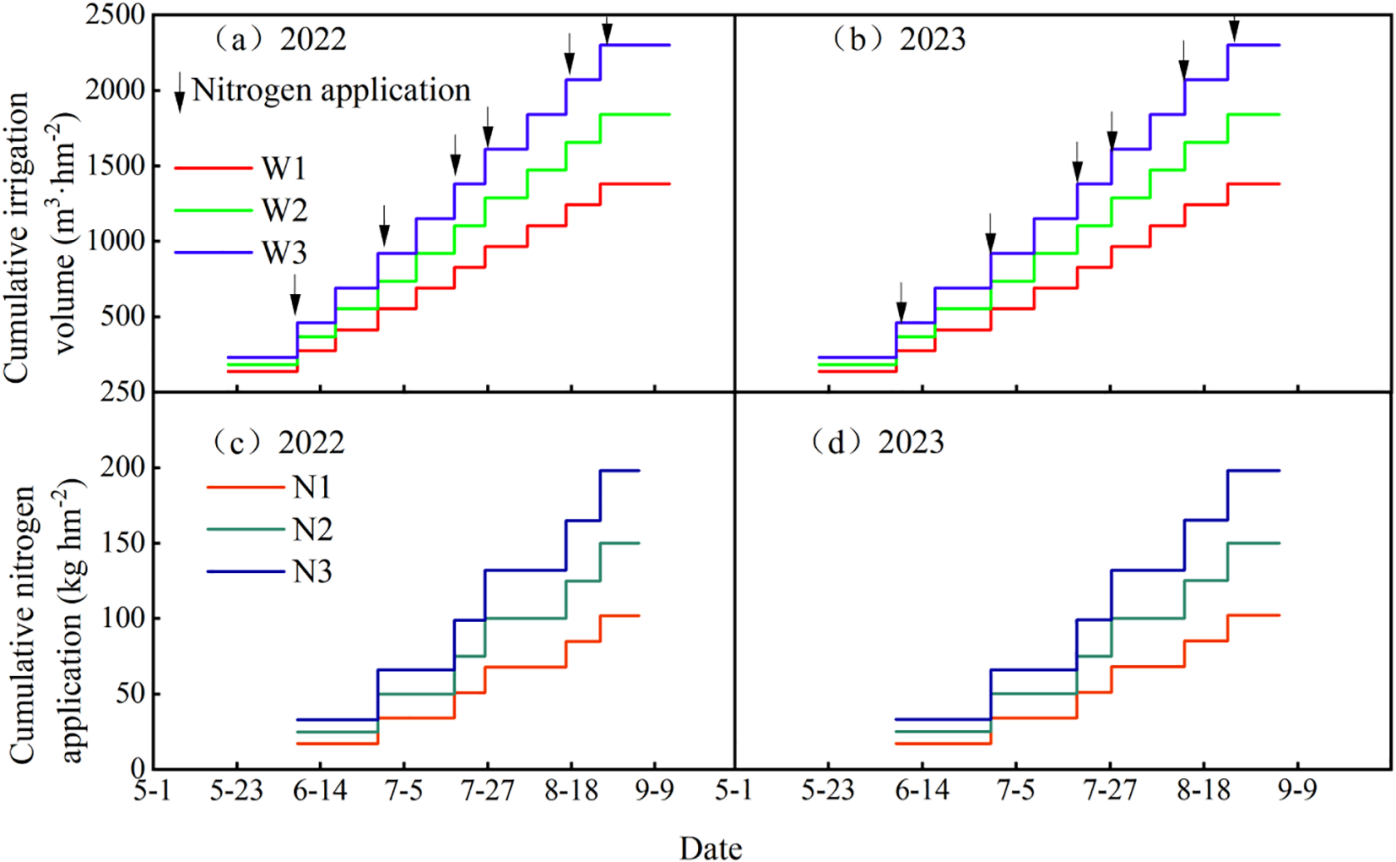
**(a–d)** Cumulative nitrogen application and irrigation amount during potato growth stages.

**Figure 3:**
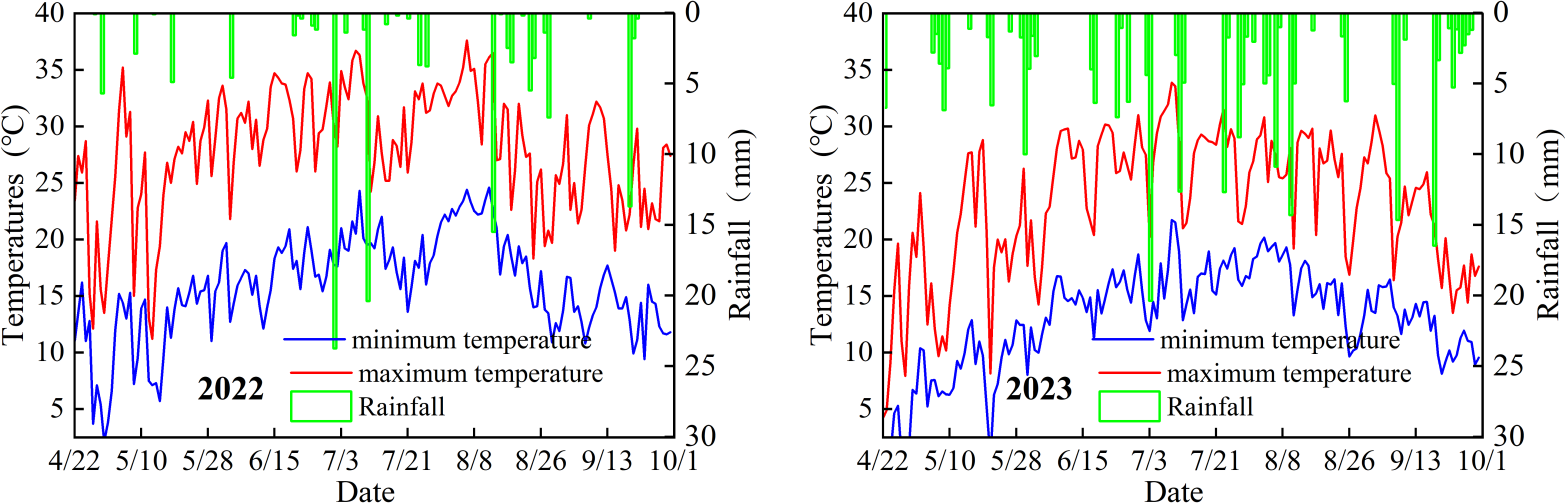
Daily average air temperature and rainfall during potato growth stages in the experimental area.

A two-year experiment was designed with two factors and three levels, involving 3 irrigation quotas and 3 nitrogen application rates, and a control group (CK) was set according to local management practices, which relied solely on rainfall without irrigation, with 15 t ha^-1^ of cow dung organic fertilizer and 195 kg ha^-1^ of nitrogen fertilizer applied. The maximum irrigation quota was implemented in accordance with the *Technical Specification for Potato Drip Irrigation Cultivation in Ningxia* issued by the Water Resources Department of Ningxia Hui Autonomous Region in 2017, while the maximum nitrogen application rate adopted the local conventional application amount. Irrigation treatments included three quotas: 2250 m^3^ ha^-1^ (W3), 20% water-saving (1800 m^3^ ha^-1^, W2), and 40% water-saving (1350 m^3^ ha^-1^, W1). Nitrogen application treatments consisted of the recommended nitrogen rate (195 kg ha^-1^, N3), 20% nitrogen reduction (156 kg ha^-1^, N2), and 40% nitrogen reduction (117 kg ha^-1^, N1), using urea (46% nitrogen content) as the nitrogen fertilizer. In total, there were 10 treatments with 3 replicates each, resulting in 30 plots, as detailed in **Figure 1b**. Irrigation and fertilization were conducted through integrated water and fertilizer management with multiple topdressings, which were applied 6 times during the seedling stage, tuber formation stage, tuber growth stage, and starch accumulation stage in a ratio of 1:2:2:1. Irrigation was carried out 10 times during the sprout growth stage, seedling stage, tuber formation stage, tuber growth stage, and starch accumulation stage in a ratio of 1:2:3:3:1, as shown in **Table 1**. The cumulative irrigation amounts and nitrogen application rates for potatoes are presented in **Figure 2**, and the rainfall and temperature during the potato growth stages are shown in **Figure 3**.

**Table 1:** Division of growth stages, irrigation amount and nitrogen application rate of potato.

Potato growth period	Sprout growth period May 10 – June 5)	Seedling stage (June 6 – June 25)	Tuber formation period (June 26 – July 25)	Tuber growth period (July 26 – August 20)	Starch accumulation period (August 21 – October 5)	Total duration
W3(m^3^ ha^-1^)	199.13	398.25	716.85	736.65	199.13	2250.00
W2(m^3^ ha^-1^)	159.30	318.60	573.48	589.32	159.30	1800.00
W1(m^3^ ha^-1^)	119.48	238.95	430.11	441.99	119.48	1350.00
N3(kg ha^-1^)	0.00	32.57	64.94	64.94	32.57	195.00
N2(kg ha^-1^)	0.00	26.05	51.95	51.95	26.05	156.00
N1(kg ha^-1^)	0.00	19.54	38.96	38.96	19.54	117.00

Potato planting dates were May 1, 2022, and April 30, 2023, with harvest dates on October 3, 2022, and October 2, 2023. The tested variety was “Qingshu 9”, which was bred by Qinghai Academy of Agricultural and Forestry Sciences. It is characterized by late-maturing and fresh-eating, luxuriant branches and leaves, concentrated tuber setting, oblong tubers, and high quality. The planting mode adopted was under-film drip irrigation, with “one film for two rows” planted at equal row spacing. The plastic film was 1.2 m wide, and the seeds were sown at a depth of 10 cm. The 1.2 m-wide plastic mulch covered seeds planted at 10 cm depth. Planting parameters included 50 cm plant spacing and 60 cm row spacing, with 10 plants per row and 40 plants per plot, resulting in a planting density of 33,345 plants ha^-1^. Basal fertilizers included calcium superphosphate (12% P_2_O_5_ content, 82.5 kg ha^-1^) and potassium sulfate (50% K_2_O content, 150 kg ha^-1^), applied as a single basal dose. Each experimental plot measured 17.6 m^2^ (5.5 m length × 3.2 m width), surrounded by 1 m-wide buffer rows between plots and 2 m-wide peripheral buffer rows. The experimental design comprised 30 plots (3 replicates × 10 treatments). Each plot featured an independent irrigation control unit with a water meter, gate valve, and pressure gauge. The embedded drip tape (16 mm inner diameter, 0.15 mm wall thickness) operated at 0.1 MPa pressure, delivering 2 L h^-1^ through emitters spaced 50 cm apart (one emitter per plant). Field management practices including weeding and pesticide application followed locally recommended agronomic protocols.

### 2.3 Sample collections and measurements

#### 2.3.1 Chlorophyll relative content (SPAD value)

The SPAD value is a parameter measured using a portable chlorophyll meter (Konica Minolta, Japan, SPAD-502Plus), which is mainly used for the rapid assessment of the relative chlorophyll content in plant leaves. Mature functional leaves from the middle part of the plant were selected—specifically the 3rd to 5th inverted leaves of potato. These leaves exhibit vigorous photosynthetic function and stable status, which can represent the overall physiological level of the plant. Additionally, intact leaves without damage were chosen to ensure data representativeness. Three to five healthy potato leaves were selected from each experimental plot for SPAD measurement, and the average value was calculated. Measurements were performed from 9:00 to 11:00 (Beijing Time) at the seedling stage, tuber formation stage, tuber bulking stage, starch accumulation stage, and late harvest stage of potato, with three replicates for each treatment.

#### 2.3.2 Leaf gas exchange parameters

Leaf gas exchange parameters were measured using a Li-6800 photosynthesis system (LI-COR, USA) to determine net photosynthetic rate (Pn), transpiration rate (Tr), stomatal conductance (Gs), and intercellular CO_2_ concentration (Ci) in potato leaves. The photosynthetic measurement system automatically recorded and stored leaf gas exchange parameters. The selection method for leaves was consistent with that used for determining relative chlorophyll content. Settings of the L-6800 instrument: The photosynthetically active radiation (PAR) in the leaf chamber was set to 1200 µmol·m^-^²·s^-^¹ to simulate normal light conditions. The reference CO_2_ concentration was set to 400 ppm (standard atmospheric concentration) for gas exchange measurements. The gas flow rate was adjusted to 500 µmol·s^-^¹ to ensure the stability of gas exchange determination. The leaf temperature was maintained at 25°C to provide optimal conditions for photosynthesis. The vapor pressure deficit (VPD) was controlled within the range of 1.0–1.5 kPa to simulate a normal transpiration environment. Measurements were conducted from 9:00 to 11:00, as light conditions are relatively stable during this period, which is suitable for photosynthesis-related gas exchange assays.

#### 2.3.3 Plant nutrient status

At the harvest stage, potato plant samples were collected, rinsed thoroughly with water, and then separated by organs (stems, leaves, roots, tubers). The samples were placed in a blast drying oven (Shanghai Yiheng Scientific Instruments Co., Ltd., DHG-9240A), blanched at 105°C for 30 minutes, and then dried to constant weight at 75°C. The dried samples were crushed using a small grinder (Tianjin Tester Instrument Co., Ltd., FW-100) and passed through a 0.5 mm sieve for the determination of total nitrogen, total phosphorus, and organic carbon contents in each organ. The TN content was measured by the Kjeldahl method (FOSS, Kjeltec 8400), the TP content by the molybdenum-antimony anti spectrophotometric method (Shanghai Yuanxi Instruments Co., Ltd., UV-5500PC), and the TOC content by the potassium dichromate titration method (Shanghai Shuli Instruments Co., Ltd., DS-101S).

#### 2.3.4 Potato yield

At potato harvest stage, randomly selected 2×2 m² plots with uniform plant growth were sampled from each treatment. Three replicated plots per treatment were cleaned with water to remove soil adhering to potato surfaces, air-dried, and weighed using an electronic balance (accuracy 0.01 g). The total yield per hectare under different treatments was calculated based on planting density.

#### 2.3.5 Leaf water use efficiency

Leaf water use efficiency (*LWUE*) of potato leaves Specific formulas see Equation 1.

(1) $$LWUE=P_{n}/T_{r}$$

Where: *LWUE* represents leaf water use efficiency (μmol mmol^-^¹), where *Pn* denotes net photosynthetic rate of potato leaves (μmol (m² s)^-^¹), and *Tr* indicates transpiration rate of potato leaves (mmol (m² s)^-^¹).

#### 2.3.6 Stoichiometric characteristics of potato plant tissues

Carbon-nitrogen-phosphorus (C:N:P) stoichiometric ratios in different potato tissues (Olivera Viciedo et al., 2019) Specific formulas see Equation 2–4.

(2) C/N=TOC/TN

(3) C/P=TOC/TP

(4) N/P=TN/TP

Where: C/N represents carbon-to-nitrogen ratio, C/P denotes carbon-to-phosphorus ratio, and N/P indicates nitrogen-to-phosphorus ratio in various tissues of potato plants.

### 2.4 TOPSIS model

The technique for order preference by similarity to ideal solution (TOPSIS) was used to identify a solution from the feasible solution set by defining the positive ideal solution and the negative ideal solution for the decision problem so that it was closest to the positive ideal solution and farthest from the negative ideal solution (Yang et al., 2023).

1. Nine treatments were set as evaluation objects, with nine evaluation indicators including tuber yield, SPAD, Tr, Pn, Gs, Ci, PSN, PLN, PRN, PTN, PSP, PLP, PRP, PTP, PSC, PLC, PRC, PTC. The evaluation indicators were normalized to establish a normalized matrix Specific formulas see Equation 5:

(5) $$z_{ij}=\frac{x_{ij}}{\sqrt{\sum_{i=1}^{n}x_{ij}^{2}}}$$

where *z_ij_* is the *j* index normalized value in *i* treatment; *x_ij_* is the *j* index value in the *i* treatment. *i* = 1, 2,···, n; *j* = 1, 2,···, m;

2. The ideal solution (*Z_ij_^+^*) and the negative solution (Z*_ij_*^−^) were determined to form the ideal solution vector *Z^+^* and the negative solution vector *Z^−^*, respectively Specific formulas see Equation 6, 7:

(6) $$Z_{ij}^{+}=\left(z_{i1}^{+},z_{i1}^{+},z_{i3}^{+}\ldots \ldots z_{ij}^{+}\right)$$

(7) $$Z_{ij}^{-}=\left(z_{i1}^{-},z_{i1}^{-},z_{i3}^{-}\ldots \ldots z_{ij}^{-}\right)$$

where *Z_ij_^+^* and *Z_ij_^−^* represent the maximum and minimum values of the evaluation object in the *j*-th index, respectively;

3. The Euclidean distances (*D_i_^+^* and *D_i_^−^*) were determined Specific formulas see Equation 8, 9:

(8) $$D_{i}^{+}=\sqrt{\sum_{j=1}^{m}{\left[w_{j}\times \left(z_{ij}-Z_{ij}^{+}\right)\right]}^{2}}$$

(9) $$D_{i}^{-}=\sqrt{\sum_{j=1}^{m}{\left[w_{j}\times \left(z_{ij}-Z_{ij}^{-}\right)\right]}^{2}}$$

where *w_j_* is the weight of indicator *j*;

4. The relative proximity coefficient *R_i_* of each treatment was calculated; that is, the proximity between the evaluation object and the optimal scheme was calculated as follows Specific formulas see Equation 10:

(10) $$R_{i}=\frac{D_{i}^{-}}{D_{i}^{+}+D_{i}^{-}}$$

### 2.5 Statistical analysis

Data collation was performed using Microsoft Excel 2019 (developed by Microsoft Corporation, USA); graphing was conducted with Origin 2021 (developed by OriginLab Corporation, USA); Duncan’s multiple comparison significance test (p< 0.05) was carried out via SPSS 26 (developed by International Business Machines Corporation, USA); and R (V.4.3.1, developed by Ross Ihaka and Robert Gentleman from the Department of Statistics, University of Auckland, New Zealand) was utilized for Pearson correlation analysis, plotting of fixed-model correlation analysis, as well as the construction of random forest and TOPSIS models.

## 3 Results and analysis

### 3.1 Effects of water-nitrogen interaction on relative chlorophyll content in potato leaves

**Figure 4:**
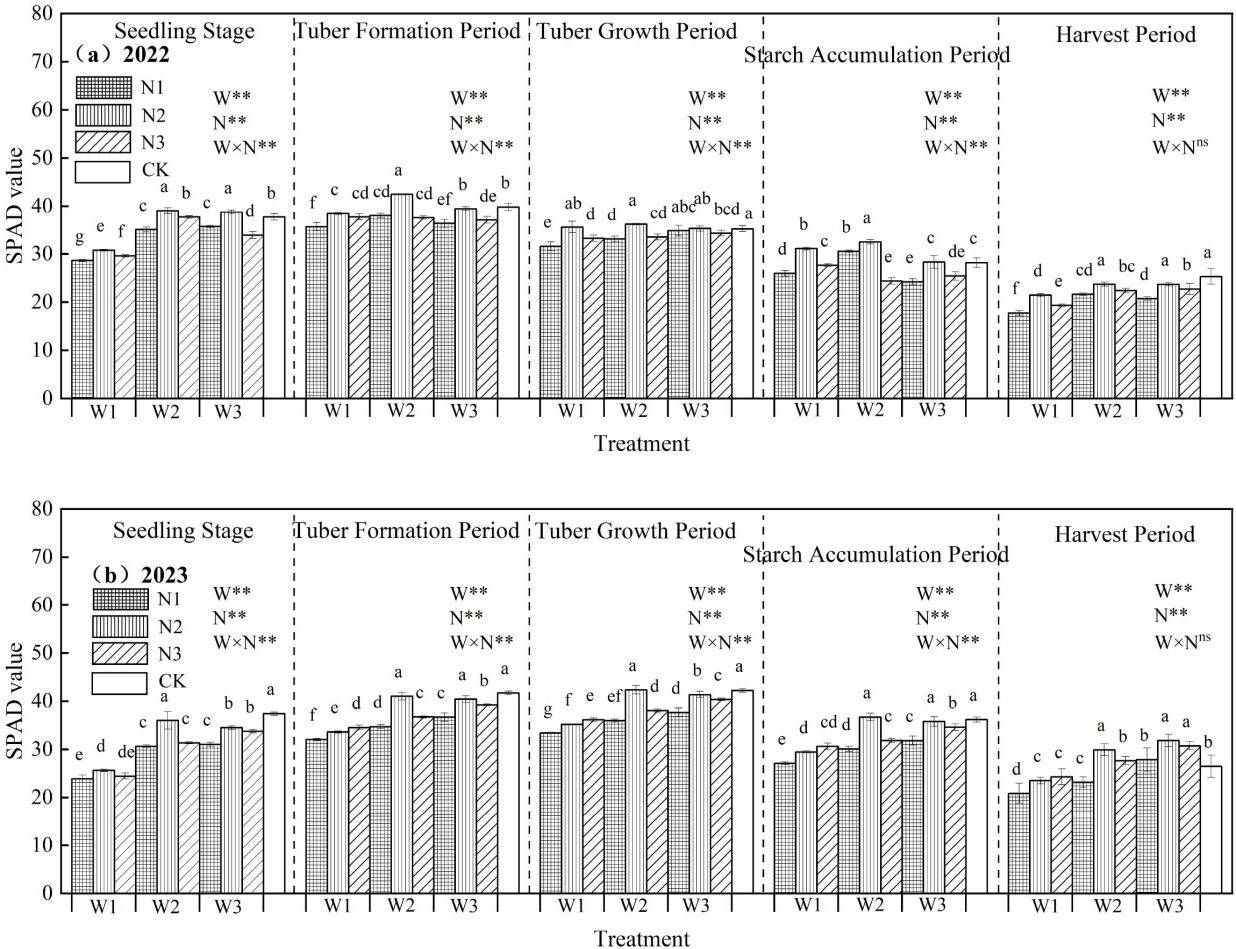
**(a, b)** Relative chlorophyll content in potato leaves under different treatments. In the figure, W stands for irrigation quota and N for nitrogen application rate. ** indicates an extremely significant effect. Different lowercase letters represent significant differences between treatments (*p* < 0.05).

The effects of water-nitrogen interaction on the relative chlorophyll content of potato leaves are shown in **Figure 4**. Consistent trends were observed across both experimental years. During the growth period, leaf SPAD values under water-nitrogen regulation exhibited an initial increase followed by a decline, reaching their maximum at the tuber formation stage. At constant nitrogen levels, SPAD values first increased and then decreased with rising irrigation quotas; similarly, under fixed irrigation, SPAD values initially rose before declining as nitrogen application rates increased. Among all treatments, the W2N2 regime resulted in the highest relative chlorophyll content, with an average increase of 2.87% compared to the CK treatment across all growth stages over both years. Statistical analysis confirmed significant interactive effects between water and nitrogen, with both factors significantly influencing leaf chlorophyll content (*p* < 0.05).

### 3.2 Effects of water-nitrogen interaction on gas exchange parameters in potato leaves

#### 3.2.1 Transpiration rate

**Figure 5:**
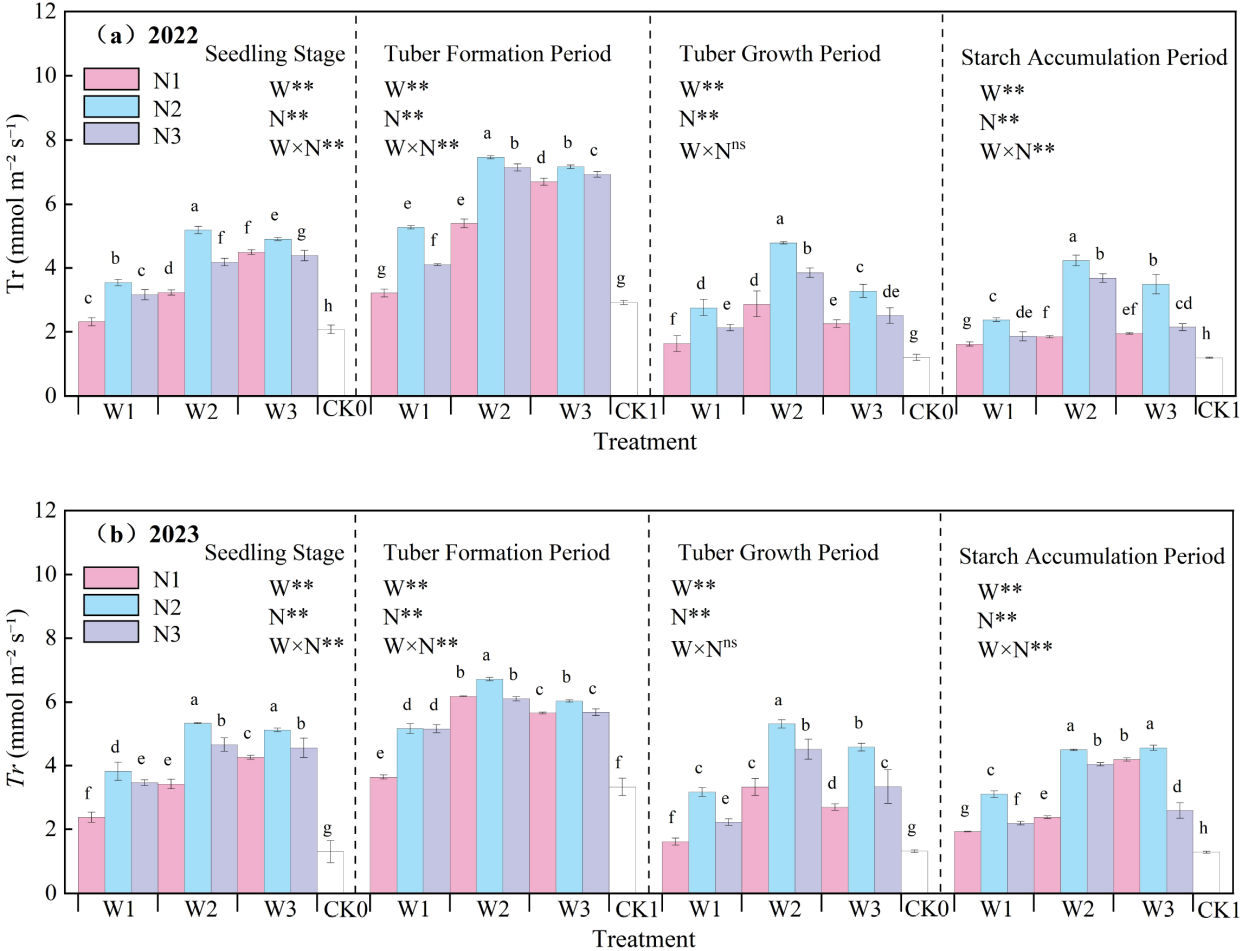
**(a, b)** Transpiration rate (*Tr*) of potato leaves. In the figure, W stands for irrigation quota and N for nitrogen application rate. ** indicates an extremely significant effect. Different lowercase letters represent significant differences between treatments (*p* < 0.05).

The effects of water-nitrogen interaction on potato leaf *Tr* are presented in **Figure 5**. Throughout the growth period, leaf transpiration generally exhibited an initial increase followed by a decline, peaking during the tuber initiation stage. Across both experimental years, under constant irrigation quotas, transpiration rates increased with rising nitrogen application up to a point, then decreased at higher nitrogen levels. Notably, the W2N2 treatment showed significantly higher *Tr* than other treatments, with an average increase of 228.49% compared to the CK treatment across all growth stages. Under fixed irrigation, the N1 treatment exhibited a steady increase in transpiration during the seedling and tuber initiation stages, whereas the N2 and N3 treatments demonstrated an initial rise followed by a decline. During the tuber bulking and starch accumulation stages, all treatments showed a consistent pattern of increase followed by decrease. The interaction between water and nitrogen reached a significant level (excluding the tuber bulking stage), and both irrigation and nitrogen application had significant effects on leaf *Tr* (Tr) (p<0.05)., underscoring that optimized irrigation and nitrogen application synergistically enhance potato leaf transpiration.

#### 3.2.2 Net photosynthetic rate

**Figure 6:**
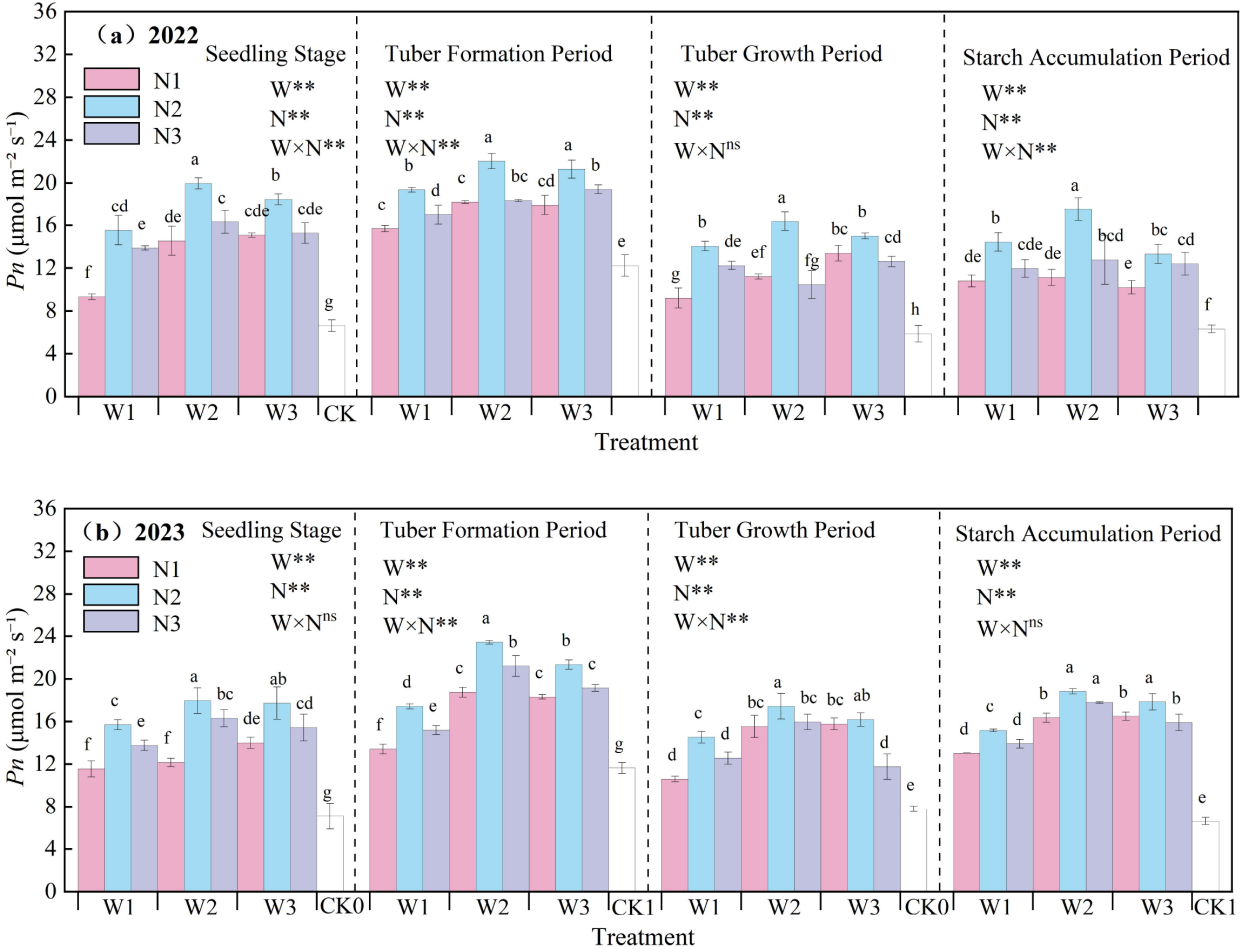
**(a, b)** Net photosynthetic rate (*Pn*) of potato leaves.

The effect of water-nitrogen interaction on the net *Pn* of potato leaves is shown in **Figure 6**. The net photosynthetic rate of potato leaves generally first increased and then decreased with the progression of growth stages, peaking at the tuber formation stage. In the two-year experiment, under the same irrigation quota, the net photosynthetic rate first increased and then decreased with the increase of nitrogen application rate. Under water-nitrogen interaction, the W2N2 treatment was significantly higher than other treatments, with an average increase of 150.09% compared to the CK treatment across all growth stages in two years. The variation trends were basically consistent in the two-year experiment. Under the same irrigation quota: for the N1 treatment, the net *Pn* increased with the increase of irrigation quota at the seedling stage and tuber bulking stage, while it first increased and then decreased at the tuber formation stage and starch accumulation stage; for the N2 treatment, it first increased and then decreased with the increase of irrigation quota at all growth stages; for the N3 treatment, it first increased and then decreased at the seedling stage and starch accumulation stage, increased at the tuber formation stage, and first decreased and then increased at the tuber bulking stage (note: corrected the repeated “tuber formation stage” in the original text). All treatments reached an extremely significant level under water-nitrogen regulation, and the significance of the water-nitrogen interaction varied among different growth stages. An appropriate irrigation quota and nitrogen application rate can improve the net photosynthetic rate of potato leaves.

#### 3.2.3 Stomatal conductance

**Figure 7:**
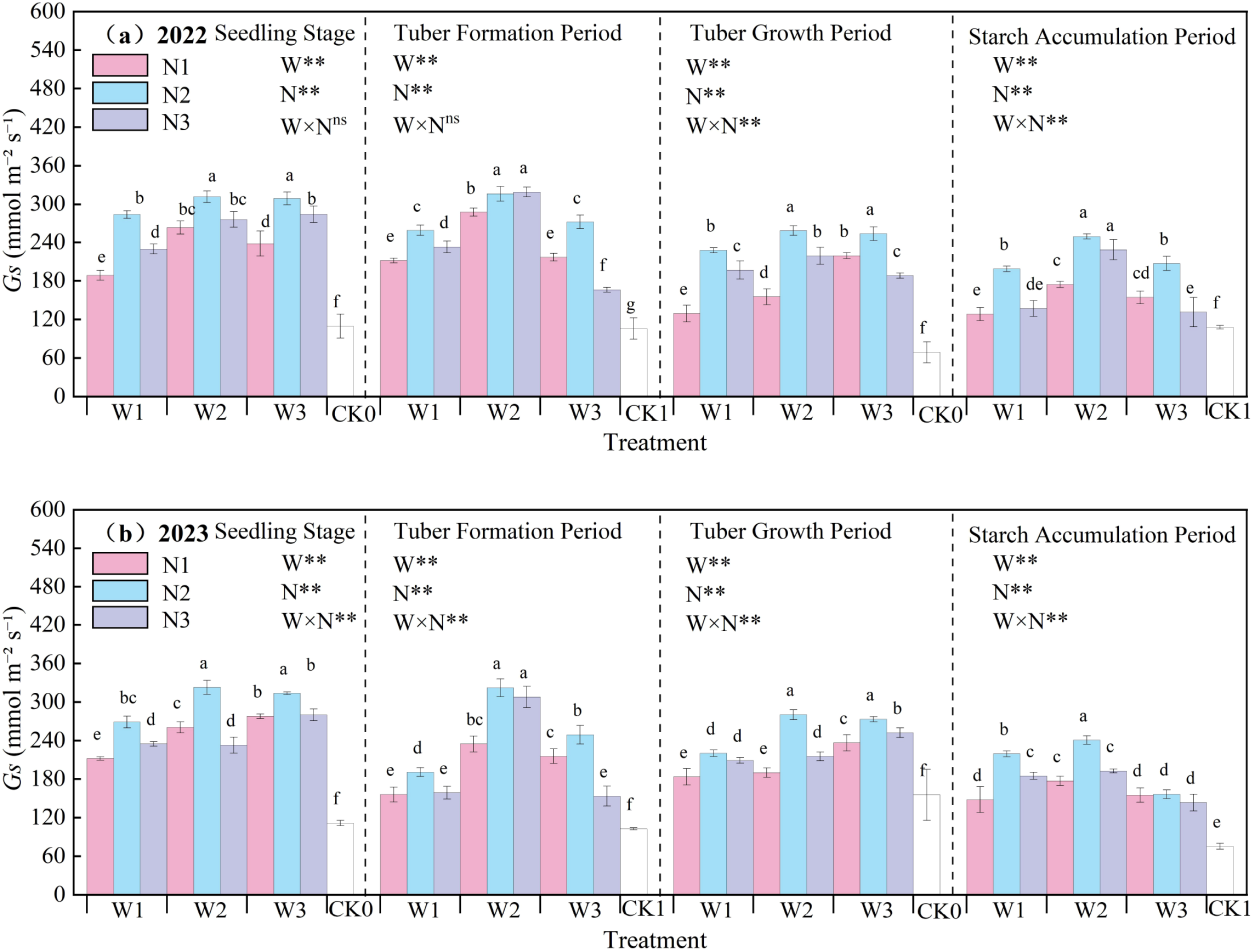
**(a, b)** Stomatal conductance (*Gs*) of potato leaves.

The effect of water-nitrogen interaction on the Gs of potato leaves is shown in **Figure 7**. The *Gs* of potato leaves generally first increased and then decreased with the progression of growth stages, peaking at the tuber formation stage. The variation trends were basically consistent in the two-year experiment: under the same irrigation quota, the leaf *Gs* first increased and then decreased with the increase of nitrogen application rate. Under water-nitrogen interaction, the W2N2 treatment was significantly higher than other treatments, with an average increase of 186.49% compared to the CK treatment across all growth stages in two years. Under the same irrigation quota, for the N1 treatment, *Gs* first increased and then decreased with the increase of irrigation quota at the seedling stage, tuber formation stage, and starch accumulation stage, and followed the same trend at the tuber bulking stage; for the N2 treatment, it first increased and then decreased with the increase of irrigation quota at all growth stages; for the N3 treatment, it increased with the increase of irrigation quota at the seedling stage, while first increasing and then decreasing at the tuber formation stage, tuber bulking stage, and starch accumulation stage. All treatments reached an extremely significant level under water-nitrogen regulation. In the second year of the experiment, the interaction between water and nitrogen had a significant effect on *Gs*. An appropriate irrigation quota and nitrogen application rate can improve the leaf *Gs* of potatoes.

#### 3.2.4 Intercellular CO_2_ concentration

**Figure 8:**
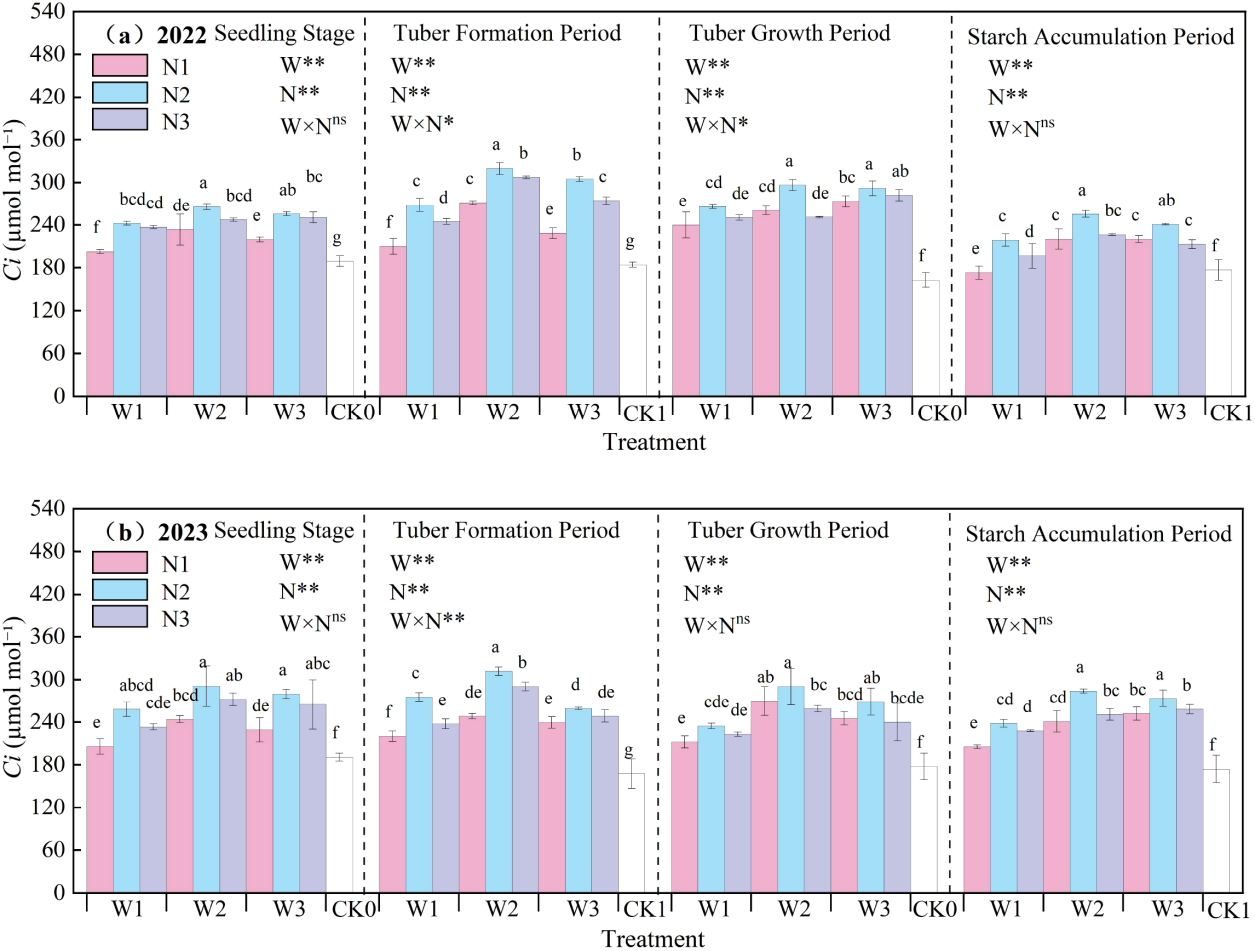
**(a, b)** Intercellular CO_2_ concentration (Ci) of potato leaves.

The effect of water-nitrogen interaction on the *Ci* of potato leaves is shown in **Figure 8**. The *Ci* of potato leaves generally first increased and then decreased with the progression of growth stages, with the maximum value observed at the tuber formation stage. In the two-year experiment, under the same irrigation quota, the leaf *Ci* first increased and then decreased with the increase of nitrogen application rate. Under water-nitrogen interaction, the W2N2 treatment was significantly higher than other treatments, with an average increase of 63.09% compared to the CK treatment across all growth stages in two years. Under the same irrigation quota, for the N1 treatment, *Ci* first increased and then decreased with the increase of irrigation quota at the seedling stage and tuber formation stage, and followed the same trend at the starch accumulation stage and tuber bulking stage; for the N2 treatment, it first increased and then decreased with the increase of irrigation quota at all growth stages; for the N3 treatment, it increased with the increase of irrigation quota at the seedling stage and tuber bulking stage, while first increasing and then decreasing at the tuber formation stage and starch accumulation stage. The interaction between water and nitrogen had a minor effect on leaf *Ci*, and no significant difference was observed at some growth stages. An appropriate irrigation quota and nitrogen application rate can improve the leaf *Ci* of potatoes.

#### 3.2.5 Leaf water use efficiency

**Figure 9:**
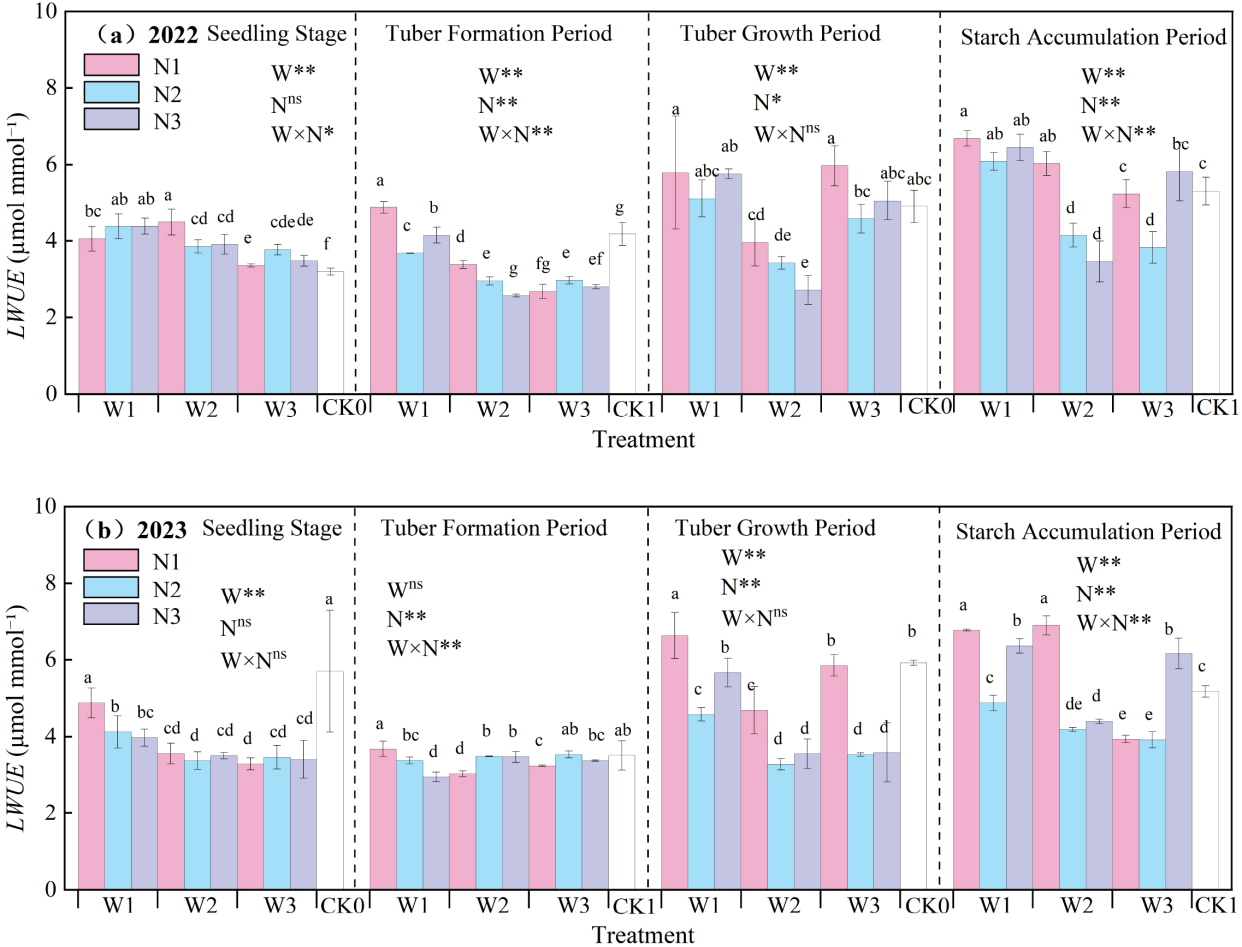
**(a, b)** Potato leaf water use efficiency (*LWUE*).

The effects of water-nitrogen interaction on potato leaf water use efficiency are shown in **Figure 9**. The *LWUE* of potato increased with the advancement of growth stages, being higher during tuber expansion and starch accumulation stages compared to seedling and tuber formation stages. Under water-nitrogen interaction, *LWUE* showed significant variations across different treatments. In 2022, the W2N1 treatment achieved the maximum value during seedling stage, representing a 40.58% increase compared to CK treatment, whereas W1N1 showed the highest values during tuber formation, tuber expansion, and starch accumulation stages due to water-nitrogen stress-induced enhancement. In 2023, the W1N1 treatment decreased by 14.44% compared to CK during seedling stage, but increased by 4.91%, 11.97%, and 30.75% during tuber formation, tuber expansion, and starch accumulation stages, respectively. These results demonstrate substantial impacts of water-nitrogen interaction on potato *LWUE*.

### 3.3 Effects of water-nitrogen interaction on potato plant nutrients

#### 3.3.1 Effects of water-nitrogen interaction on organic carbon content in potato plants

Plant organic carbon refers to the carbon element contained in organic matter within plant tissues. The effects of water-nitrogen interaction on organic carbon content in various organs of potato plants are shown in **Table 2**. Under water-nitrogen treatments, the organic carbon content trends across potato plant organs were generally consistent. Under the same irrigation quota, organic carbon content in all organs increased with higher nitrogen application rates. Under the same nitrogen application rate, organic carbon content increased with higher irrigation quotas. However, the interaction between the two factors did not reach a significant level, likely due to the relatively stable variation in organic carbon content.

**Table 2:** Organic carbon content in potato plants under different treatments.

Treatment	Stem(g kg^-1^)		Leaf(g kg^-1^)		Root(g kg^-1^)		Tubers(g kg^-1^)	
	2022	2023	2022	2023	2022	2023	2022	2023
W1N1	351.70a	291.32a	305.76a	325.78b	314.56b	305.02d	302.60a	307.49a
W1N2	352.03a	296.32a	324.98a	332.79b	329.06b	345.22cd	308.07a	310.40a
W1N3	355.55a	307.12a	325.26a	338.45b	366.30ab	374.98abc	319.23a	327.75a
W2N1	362.44a	315.16a	347.41a	340.87b	371.80ab	350.94cd	333.58a	335.20a
W2N2	365.00a	319.58a	353.23a	345.92b	372.24ab	368.6bc	339.22a	341.67a
W2N3	370.89a	325.38a	357.57a	346.02b	378.93ab	404.64abc	337.18a	341.91a
W3N1	371.91a	324.68a	360.26a	380.95b	401.08a	383.16abc	320.01a	322.08a
W3N2	374.54a	331.78a	364.06a	432.37a	404.53a	425.58ab	330.84a	332.53a
W3N3	379.54a	352.83a	362.00a	460.18a	419.90a	433.61a	356.4a	360.55a
CK	346.70a	286.35a	291.88a	314.67c	310.90b	260.66e	300.17a	305.38a
W	ns	ns	*	**	*	*	ns	ns
N	ns	ns	ns	ns	ns	ns	ns	ns
W×N	ns	ns	ns	ns	ns	ns	ns	ns

#### 3.3.2 Effects of water-nitrogen interaction on total nitrogen content in potato plants

The effect of water-nitrogen interaction on the total nitrogen content of various potato plant organs is shown in **Table 3**. The two-year experimental results indicated that the variation trends of all treatments were basically consistent with the change of water-nitrogen interaction. Under the same irrigation quota, the total nitrogen content of potato stems first increased and then decreased with the increase of nitrogen application rate. Under the same nitrogen application rate, with the increase of irrigation quota, the total nitrogen content increased with the increase of nitrogen application rate in the N1 treatment, while it first increased and then decreased in the N2 and N3 treatments. Among all treatments, the W2N2 treatment achieved the highest value, with an average increase of 55.61% compared to the CK treatment over two years. Under the same irrigation quota, the total nitrogen content of potato tubers first increased and then decreased with the increase of nitrogen application rate. Under the same nitrogen application rate, in 2022, the total nitrogen content increased with the increase of irrigation quota in the N1 treatment, while it first increased and then decreased in the N2 and N3 treatments; in 2023, the total nitrogen content of all treatments first decreased and then increased with the increase of irrigation quota. The W2N2 treatment had the highest value, with an average increase of 61.02% compared to the CK treatment over two years. Under the same irrigation quota, the total nitrogen content of potato roots first increased and then decreased with the increase of nitrogen application rate. Under the same nitrogen application rate, the total nitrogen content of all treatments first decreased and then increased with the increase of irrigation quota. The W2N2 treatment showed the highest value, with an average increase of 90.95% compared to the CK treatment over two years. Across the two-year experiment, the W2N2 treatment had the highest total nitrogen content of potato leaves under water-nitrogen interaction, with an average increase of 121.91% compared to the CK treatment. The interaction between water and nitrogen had a significant effect on the total nitrogen content of potato leaves.

**Table 3:** Total nitrogen content in potato plants under different treatments.

Treatment	Stem(g kg^-1^)		Leaf(g kg^-1^)		Root(g kg^-1^)		Tubers(g kg^-1^)	
	2022	2023	2022	2023	2022	2023	2022	2023
W1N1	7.66d	8.03f	22.6f	23.37d	9.22g	13.23e	9.89h	11.98f
W1N2	10.07bc	11.34c	30.65c	27.45b	12.02e	15.76c	12.75e	14.49d
W1N3	7.4cd	9.63d	27.1d	26.06c	11.42f	13.82de	11.89f	13.65e
W2N1	10.29ab	8.97e	22.58f	31.86a	16.99cd	14.12d	10.92g	12.85e
W2N2	12.69a	13.18a	34.17a	33.12a	19.21a	20.71a	17.06a	18.74a
W2N3	11.51ab	13.31a	33.95a	32.35a	16.48d	17.51b	16.32b	18.2a
W3N1	11.34ab	9.39de	24.87e	31.74a	17.12c	14.33d	11.07g	13.15e
W3N2	9.78bc	12.08b	32.59b	32.69a	18.56b	14.62d	15.3c	17.16b
W3N3	10.98ab	11.42c	31.67bc	31.62a	18.47b	14.75d	13.73d	15.74c
CK	7.21d	8.11f	20.26g	21.24d	9.20g	11.26e	6.97i	8.73f
W	*	**	**	**	**	**	**	**
N	ns	**	**	**	**	**	**	**
W×N	ns	**	**	**	**	**	**	**

#### 3.3.3 Effects of water-nitrogen interaction on total phosphorus content in potato plants

The effects of water-nitrogen interaction on the total phosphorus content in various organs of potato plants are shown in **Table 4**. Results from the two-year experiment indicated that the variation trends of all treatments under water-nitrogen interaction were generally consistent. Under the same irrigation quota, for the W1 treatment, the total phosphorus content in potato stems showed an increasing trend with the increase of nitrogen application rate; while for the W2 and W3 treatments, it first increased and then decreased. Under the same nitrogen application rate, the total phosphorus content in potato stems first increased and then decreased with the increase of nitrogen application rate. Among them, the W2N2 treatment was the highest, with an average increase of 80.94% compared with the CK treatment over the two years. Under the same irrigation quota, the total phosphorus content in potato stems first increased and then decreased as the nitrogen application rate increased. Under the same nitrogen application rate, with the increase of irrigation quota, the total phosphorus content in potato stems also showed a trend of first increasing and then decreasing. The W2N2 treatment was the maximum in this scenario, with an average increase of 35.36% compared with the CK treatment over the two years. Under the same irrigation quota, for the W1 treatment, the total phosphorus content in potato stems increased with the increase of nitrogen application rate; for the W2 and W3 treatments, the total phosphorus content in potato stems first increased and then decreased as the nitrogen application rate increased. Under the same nitrogen application rate, all treatments first decreased and then increased with the increase of irrigation quota. The W2N2 treatment was the highest, with an average increase of 102.11% compared with the CK treatment. Overall, the two-year experimental results showed that under water-nitrogen interaction, the W2N2 treatment had the highest total phosphorus content, with an average increase of 34.34% compared with the CK treatment over the two years.

**Table 4:** Total phosphorus content of potato plants under different treatments.

Treatment	Stem(g kg^-1^)		Leaf(g kg^-1^)		Root(g kg^-1^)		Tubers(g kg^-1^)	
	2022	2023	2022	2023	2022	2023	2022	2023
W1N1	1.24g	0.87f	2.46f	1.59d	1.21f	0.58a	2.05g	2.18h
W1N2	1.27f	0.98e	2.64e	1.81bc	1.25f	0.87a	2.23de	2.37ef
W1N3	1.33e	1.18d	2.57ef	1.69cd	1.46e	0.94a	2.13f	2.27g
W2N1	1.74b	1.16d	3.12c	1.76bc	1.62cd	1.12a	2.2e	2.35f
W2N2	1.91a	1.6a	3.43a	2.04a	1.88a	1.45a	2.66a	2.81a
W2N3	1.74b	1.33c	3.29ab	1.78bc	1.68bc	1.22a	2.6b	2.74b
W3N1	1.52d	1.19d	2.88d	1.82bc	1.57d	1.01a	2.25d	2.4de
W3N2	1.75b	1.41b	3.14bc	2.00a	1.76b	1.19a	2.56c	2.69c
W3N3	1.68c	1.30c	2.99cd	1.89ab	1.65c	1.1a	2.25d	2.41d
CK	1.20g	0.79f	2.46f	1.56d	1.14f	0.55b	1.95h	2.10h
W	**	**	**	**	**	**	**	**
N	**	**	**	**	**	**	**	**
W×N	**	**	ns	ns	**	ns	**	**

#### 3.3.4 Distribution ratio of nutrient content in different organs of potato plants

**Figure 10:**
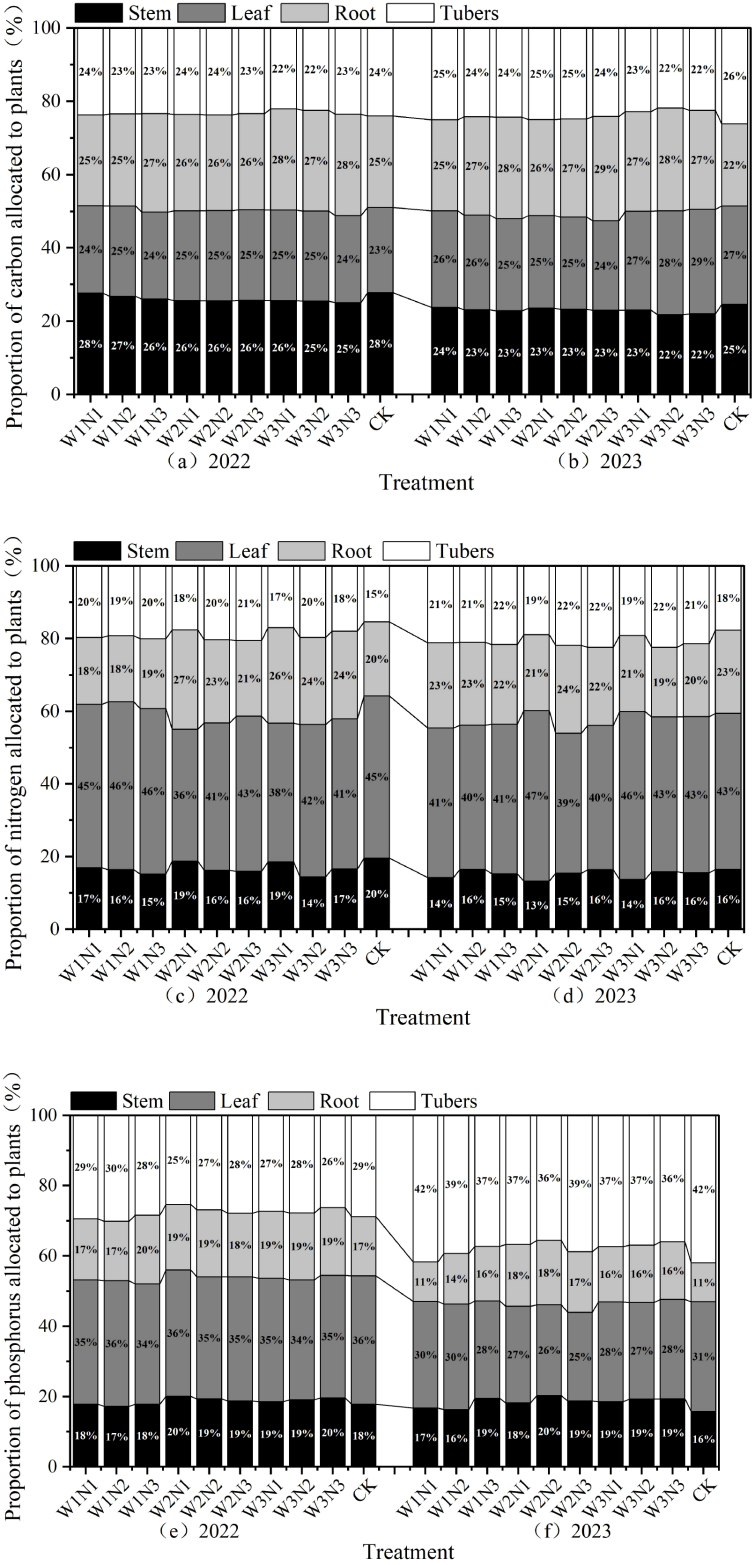
**(a–f)** Distribution ratios of nutrient content in different organs of potato plants.

The distribution ratios of organic carbon content in various organs of potato plants in 2022 are shown in **Figure 10a**. Under water-nitrogen regulation in 2022, the organic carbon content in potato stems and roots was greater than that in leaves and tubers. In the W1N1, W1N2, W1N3, W2N1, W2N2, and W2N3 treatments, the organic carbon content followed the order: stem > root > leaf > tuber. In the W3N1, W3N2, and W3N3 treatments, the order was root > stem > leaf > tuber. In 2023, the W1N1 and W3N3 treatments showed organic carbon content in the order: leaf > root > tuber > stem, while the W1N2, W1N3, W2N1, W2N2, W2N3, W3N1, and W3N2 treatments followed root > leaf > tuber > stem. This indicates that water-nitrogen interaction can improve the organic carbon content distribution among potato plant organs.

The distribution ratios of total nitrogen content in various organs of potato plants are shown in **Figure 10b**. Under water-nitrogen interaction in 2022, stem total nitrogen accounted for 13%~16% of the total, leaf total nitrogen for 39%~47%, root total nitrogen for 19%~24%, and tuber total nitrogen for 18%~22%, with leaves exhibiting the highest total nitrogen content. Under water-nitrogen regulation, the W1N1, W1N2, and W1N3 treatments showed total nitrogen content in the order: leaf > tuber > root > stem, while the W2N1, W2N2, W2N3, W3N1, W3N2, and W3N3 treatments followed leaf > root > tuber > stem. In 2023, stem total nitrogen accounted for 13%~18%, leaf total nitrogen for 39%~45%, root total nitrogen for 21%~25%, and tuber total nitrogen for 17%~22%, with leaves and tubers showing the highest total nitrogen content. Under water-nitrogen regulation, all treatments exhibited total nitrogen content in the order: leaf > root > tuber > stem. This indicates that water-nitrogen interaction can improve total nitrogen content distribution among potato plant organs.

The distribution ratios of total phosphorus content in various organs of potato plants are shown in **Figure 10c**. Under water-nitrogen interaction in 2022, stem total phosphorus accounted for 18%~20%, leaf total phosphorus for 34%~36%, root total phosphorus for 17%~20%, and tuber total phosphorus for 25%~30%, with leaves exhibiting the highest total phosphorus content. Under water-nitrogen regulation, the W1N1, W2N1, W2N2, W2N3, W3N1, W3N2, and W3N3 treatments showed total phosphorus content in the order: leaf > tuber > stem > root, while the W1N2 and W1N3 treatments followed leaf > tuber > root > stem. In 2023, stem total phosphorus accounted for 16%~20%, leaf total phosphorus for 27%~31%, root total phosphorus for 11%~18%, and tuber total phosphorus for 35%~42%, with leaves exhibiting the highest total phosphorus content. Under water-nitrogen regulation, all treatments exhibited total phosphorus content in the order: tuber > leaf > stem > root. This indicates that water-nitrogen interaction can improve total phosphorus content distribution among potato plant organs.

### 3.4 Effects of water-nitrogen interaction on stoichiometric characteristics of potato plants

#### 3.4.1 Carbon-nitrogen stoichiometric ratios in different potato plant parts

The C:N stoichiometric ratios of different parts of potato plants under various water-nitrogen interaction treatments are shown in **Table 5**. The variation trends over the two-year experimental period were generally consistent. Under the same irrigation quota, the C:N ratio of stems showed an initial decrease followed by an increase with increasing nitrogen application. Under the same nitrogen application rate, the C:N ratio exhibited an initial decrease then increase with increasing irrigation quota. Compared to the CK treatment, the W1N1 treatment increased by 3.58%, while the W1N2, W1N3, W2N1, W2N2, W2N3, W3N1, W3N2, and W3N3 treatments decreased by 22.02%, 4.27%, 11.62%, 32.03%, 28.44%, 13.02%, 18.59%, and 18.65%, respectively. The C:N ratios of leaves in the W1N1, W1N2, W1N3, W2N1, W2N2, W2N3, W3N1, W3N2, and W3N3 treatments decreased by 6.03%, 22.21%, 14.50%, 10.69%, 28.86%, 27.31%, 9.29%, 16.45%, and 10.97%, respectively, compared to CK. The C:N ratios of roots in the W1N1, W1N2, W1N3, W2N1, W2N2, W2N3, W3N1, W3N2, and W3N3 treatments increased by 65.84%, 61.48%, 68.56%, 17.53%, 25.89%, 47.33%, 55.66%, 51.66%, and 35.82%, respectively, compared to CK. The C:N ratios of tubers in the W1N1, W1N2, W1N3, W2N1, W2N2, W2N3, W3N1, W3N2, and W3N3 treatments increased by 21.00%, 12.04%, 23.05%, 13.48%, 5.03%, 12.29%, 3.54%, 41.68%, respectively, compared to CK, while the W2N2 treatment decreased by 10.03%.

**Table 5:** Carbon-nitrogen stoichiometric ratios in different parts of potato plants.

Treatment	Stem		Leaf		Root		Tubers	
	2022	2023	2022	2023	2022	2023	2022	2023
W1N1	41.56a	36.24a	13.5bc	13.92ab	34.05a	23.03cd	30.57ab	25.63a
W1N2	32.47bc	26.1e	10.59d	12.11c	27.34b	21.87d	24.14a	21.4b
W1N3	40.06a	31.84bc	11.98cd	12.97bc	32.04a	27.11ab	26.81abcd	23.98b
W2N1	31.28bcd	35.1ab	15.37a	10.69d	21.85cd	24.8bc	30.51cd	26.04d
W2N2	26.85e	24.2e	10.33d	10.43d	19.36d	17.78e	19.87e	18.23e
W2N3	29.34de	24.41e	10.52d	10.69d	22.98c	23.09cd	20.65d	18.77cd
W3N1	30.81cd	34.52abc	14.48ab	11.99c	23.42c	26.7ab	28.87abc	24.46b
W3N2	33.73b	27.42de	11.16d	13.22bc	21.78cd	29.07a	21.59bcd	19.36bc
W3N3	30.22cd	30.88cd	11.43d	14.55a	22.72cd	29.36a	25.91abcd	22.88b
CK	39.92a	35.19ab	14.39a	14.79a	33.72a	23.08cd	42.95a	34.94a
W	**	*	ns	**	**	**	**	**
N	**	**	**	ns	**	**	**	**
W×N	**	*	ns	*	ns	**	*	**

#### 3.4.2 Carbon-phosphorus stoichiometric ratios in different potato plant parts

The carbon and nitrogen stoichiometric ratios in various parts of potato plants under different water-nitrogen interaction treatments are shown in **Table 6**. The variation trends over the two-year experimental period were generally consistent. The C:P ratios of stems in the W1N1, W1N2, W1N3, W2N1, W2N2, W2N3, W3N1, W3N2, and W3N3 treatments decreased by 5.25%, 10.98%, 19.05%, 26.35%, 39.96%, 29.54%, 20.54%, 30.90%, and 23.42%, respectively, compared to the CK treatment. The C:P ratios of leaves in the W1N1, W1N3, W3N1, W3N2, and W3N3 treatments increased by 3.07%, 2.31%, 4.44%, 3.73%, and 14.02%, respectively, while the W1N2, W2N1, W2N2, and W2N3 treatments decreased by 3.91%, 4.54%, 14.72%, and 5.22%, compared to CK. The C:P ratios of roots in the W1N1, W1N2, W1N3, W2N1, W2N2, W2N3, W3N1, W3N2, and W3N3 treatments increased by 140.05%, 141.37%, 105.65%, 76.70%, 93.44%, 121.18%, 124.63%, 128.56%, and 164.79%, respectively, compared to CK. The C:P ratios of tubers in the W1N1, W1N2, W1N3, W2N1, W2N2, W2N3, W3N1, W3N2, and W3N3 treatments increased by 80.93%, 83.39%, 89.81%, 77.07%, 63.54%, 71.35%, 72.47%, 80.92%, and 96.79%, respectively, compared to CK.

**Table 6:** Carbon: Phosphorus (C:P) stoichiometric ratios in different tissues of potato plants.

Treatment	Stem		Leaf		Root		Tubers	
	2022	2023	2022	2023	2022	2023	2022	2023
W1N1	283.18a	332.67a	124.25a	205.04bcd	259.49ab	520.99a	147.77ab	140.91abc
W1N2	276.78ab	301.8ab	123.02ab	183.97e	262.67a	396.44b	138.23ab	131.14abc
W1N3	266.79ab	259.35cd	126.52a	200.34cd	249.98abcd	396.87b	150.08ab	144.47ab
W2N1	208.06cd	270.63bc	111.29abc	193.69de	229.44cd	311.95d	151.43ab	142.51abc
W2N2	191.18d	199.08e	102.83c	169.6f	197.52e	253.4e	127.55b	121.52c
W2N3	212.7cd	245.26cd	108.44bc	194.34de	224.97d	331.05cd	129.77b	124.55bc
W3N1	244.05bc	272.41bc	124.78a	208.88bc	255.82abc	379.4b	142.38ab	134.36abc
W3N2	213.59cd	235.55d	115.86abc	215.54b	230.07bcd	357.97bc	129.22b	123.48bc
W3N3	225.65cd	272.1bc	120.9ab	243.37a	253.7abcd	394.46b	158.28a	149.67a
CK	288.55a	361.42a	118.36ab	201.11cd	273.25a	469.84a	154.15a	145.07a
W	**	**	**	**	**	**	ns	ns
N	ns	**	ns	**	*	**	*	*
W×N	ns	**	ns	**	ns	**	ns	ns

#### 3.4.3 Nitrogen: phosphorus stoichiometric ratios in different tissues of potato plants

Under different water-nitrogen interaction treatments, the carbon-nitrogen stoichiometric ratios of various parts of potato plants are shown in **Table 7**. The carbon-nitrogen stoichiometric ratio of potato stems in treatments W1N1, W1N3, W2N1, W2N2, W2N3, W3N1, W3N2, and W3N3 changed by 8.97%, 15.44%, 18.27%, 12.77%, 1.70%, 10.16%, 15.04%, and 7.21% compared to the CK treatment, respectively, while the W1N2 treatment changed by 14.07% compared to CK. The carbon-nitrogen stoichiometric ratio of potato leaves in treatments W1N1, W1N2, W1N3, W2N1, W2N2, W2N3, W3N1, W3N2, and W3N3 increased by 9.52%, 22.75%, 19.04%, 16.16%, 20.05%, 30.48%, 19.31%, 22.20%, and 25.13% compared to the CK treatment, respectively. The carbon-nitrogen stoichiometric ratio of potato roots in treatments W1N1, W1N2, W1N3, W2N1, W2N2, W2N3, W3N1, W3N2, and W3N3 increased by 218.02%, 199.75%, 151.48%, 205.43%, 203.09%, 196.30%, 187.04%, 196.17%, and 289.51% compared to the CK treatment, respectively. The carbon-nitrogen stoichiometric ratio of potato tubers in treatments W1N1, W1N2, W1N3, W2N1, W2N2, W2N3, W3N1, W3N2, and W3N3 increased by 205.87%, 227.93%, 208.94%, 224.86%, 264.53%, 228.77%, 215.92%, 249.72%, and 186.31% compared to the CK treatment, respectively.

**Table 7:** Nitrogen: Phosphorus (N:P) stoichiometric ratios in different tissues of potato plants.

Treatment	Stem		Leaf		Root		Tubers	
	2022	2023	2022	2023	2022	2023	2022	2023
W1N1	6.85bc	9.19c	9.2d	14.73e	7.62e	22.67a	4.83e	5.49d
W1N2	8.54a	11.56a	11.62a	15.2de	9.62d	18.14b	5.72d	6.12bc
W1N3	6.76bc	8.14e	10.56b	15.45d	7.8e	14.66c	5.59d	6.02c
W2N1	6.69bc	7.71f	7.24f	18.14a	10.49b	12.57e	4.96e	5.47d
W2N2	7.14bc	8.23e	9.96c	16.27c	10.2c	14.25c	6.42a	6.67a
W2N3	7.28abc	10.04b	10.31b	18.2a	9.79d	14.35c	6.28ab	6.63a
W3N1	7.93ab	7.9f	8.63e	17.44b	10.94a	14.21c	4.93e	5.49d
W3N2	6.39c	8.58d	10.39b	16.31c	10.56b	12.31e	5.98c	6.38ab
W3N3	7.54abc	8.81d	10.6b	16.74c	11.18a	13.43d	6.1bc	6.54a
CK	7.35abc	10.27b	8.24e	13.61e	8.1e	20.37a	3.58f	4.15d
W	ns	**	**	**	**	**	**	**
N	ns	**	**	**	**	**	**	**
W×N	**	**	**	**	**	**	**	*

### 3.5 Effects of water-nitrogen interaction on potato yield

**Figure 11:**
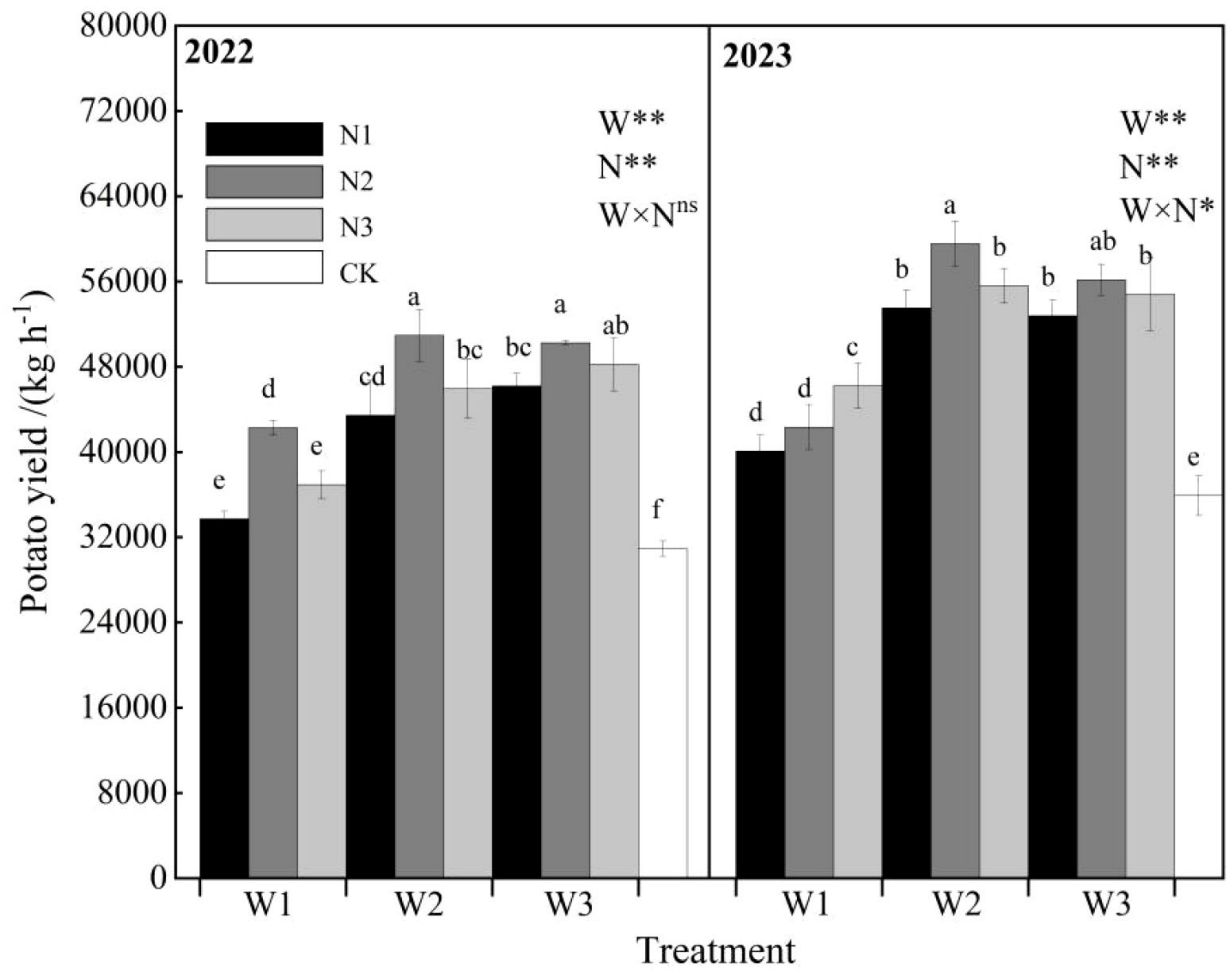
Effects of water-nitrogen interaction on potato yield.

The interactive effects of water and nitrogen on total potato yield are shown in **Figure 11**. The variation trends of total potato yield under water-nitrogen regulation were generally consistent across the two experimental years. In 2022, under the same irrigation quota, total yield initially increased and then decreased with increasing nitrogen application rate. Under the same nitrogen application rate, total yield showed increasing trends with higher irrigation quotas for N1 and N3 treatments, while N2 treatment exhibited an initial increase followed by decrease. Compared with CK treatment, W1N1, W1N2, W1N3, W2N1, W2N2, W2N3, W3N1, W3N, and W3N3 increased yields by 9.03%, 36.67%, 19.39%, 40.43%, 64.71%, 48.53%, 49.33%, 62.45%, and 55.90% respectively. In 2023, under W1 treatment, total yield increased continuously with nitrogen application, while W2 and W3 treatments showed an initial increase followed by decrease. Under the same nitrogen application rate, all treatments exhibited an initial increase followed by decrease in total yield with increasing irrigation quota. Compared with CK treatment, W1N1, W1N2, W1N3, W2N1, W2N2, W2N3, W3N1, W3N, and W3N3 increased yields by 11.62%, 17.88%, 28.70%, 49.05%, 65.84%, 54.81%, 46.98%, 56.37%, and 52.62% respectively. Excessive irrigation and nitrogen application were detrimental to yield formation, while water and nitrogen demonstrated synergistic effects in enhancing potato productivity.

### 3.6 Correlation between potato yield and key indicators under water-nitrogen interaction and optimization of water-nitrogen regimes

**Figure 12:**
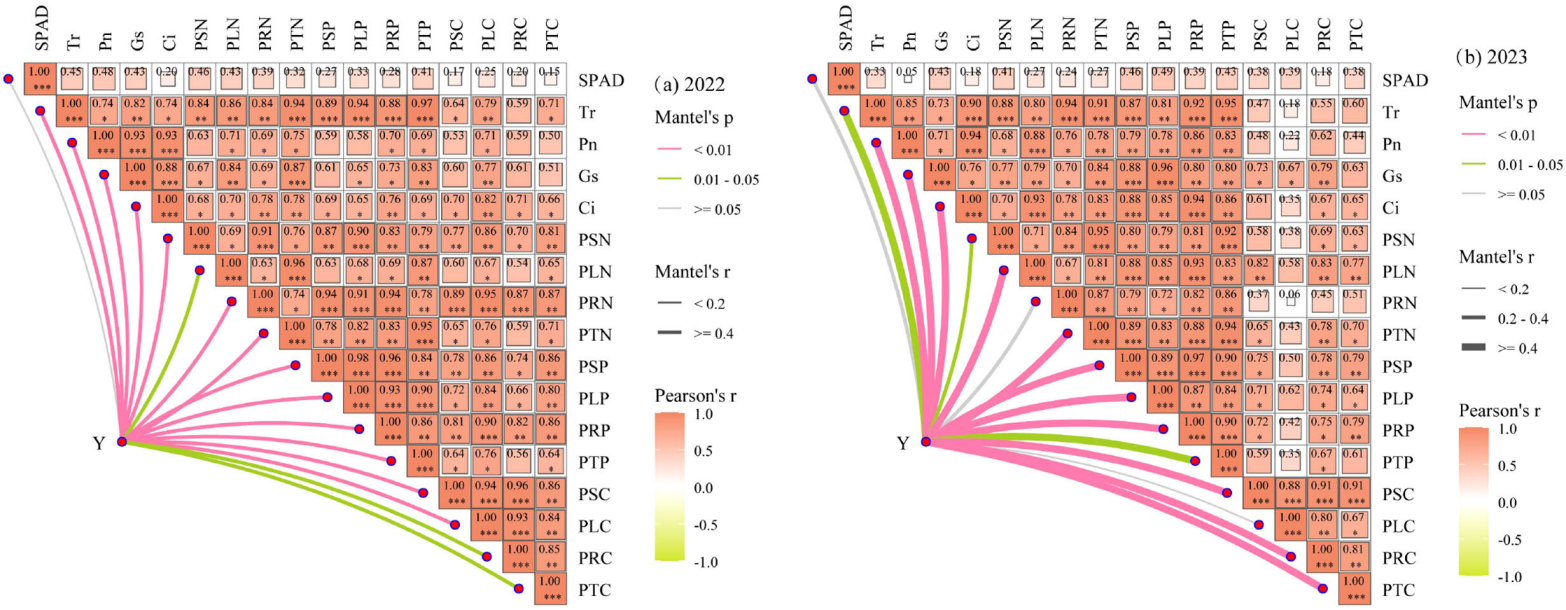
Correlation between potato yield and various indicators. *P < 0.05, **P < 0.01, ***P < 0.001.

The physiological ecosystem of plant leaves exhibits significant spatial variability, and it is difficult for a single process of changes in potato physiological indicators and plant nutrients to reflect the scientific nature of potato growth. To further clarify the regulatory mechanism of water-nitrogen interaction on the photosynthetic stoichiometric balance of potatoes and the relationship between potato yield and various indicators, a Mantel test analysis was conducted on each potato indicator and potato yield (Y) (**Figure 12**). Since the potato tuber growth stage is the most critical period for yield formation, the correlation analysis was performed using the average values of the potato tuber growth stage. As shown in **Figure 12a**, the results of Mantel test analysis on soil indicators and potato yield in 2022 indicated that the overlap between SPAD index and potato yield was poor (Mantel r< 0.4), suggesting that the SPAD index had little impact on yield. Other indicators showed good overlap with potato yield (*Mantel r ≥* 0.4) and had a strong correlation with potato yield (Mantel P ≤ 0.05), indicating that Tr, Pn, Gs, Ci, PSN, PLN, PRN, PTN, PSP, PLP, PRP, PTP, PSC, PLC, PRC, and PTC all had significant impacts on potato yield. As can be seen from **Figure 12b**, in 2023, the overlap between potato leaf SPAD, PRN, PLC indicators and potato yield was poor (*Mantel r<* 0.2), implying that SPAD, PRN, and PLC had little influence on potato yield. Except for soil Tr, PSN, and PTP, which had a weak correlation with potato yield (Mantel P< 0.05), other soil indicators showed a strong correlation with potato yield (Mantel P ≤ 0.05). There were correlations among various indicators of potato plants (except SPAD), indicating that there was a promoting effect between the physiological indicators of the plant. Therefore, Pearson correlation analysis clarified that there were strong correlations among various soil indicators under water-nitrogen interaction, and also illuminated the correlations among SPAD, leaf photosynthesis, and plant nutrients, providing a reference for clarifying the regulatory mechanism of water-nitrogen interaction on the photosynthetic stoichiometric balance of potatoes.

**Figure 13:**
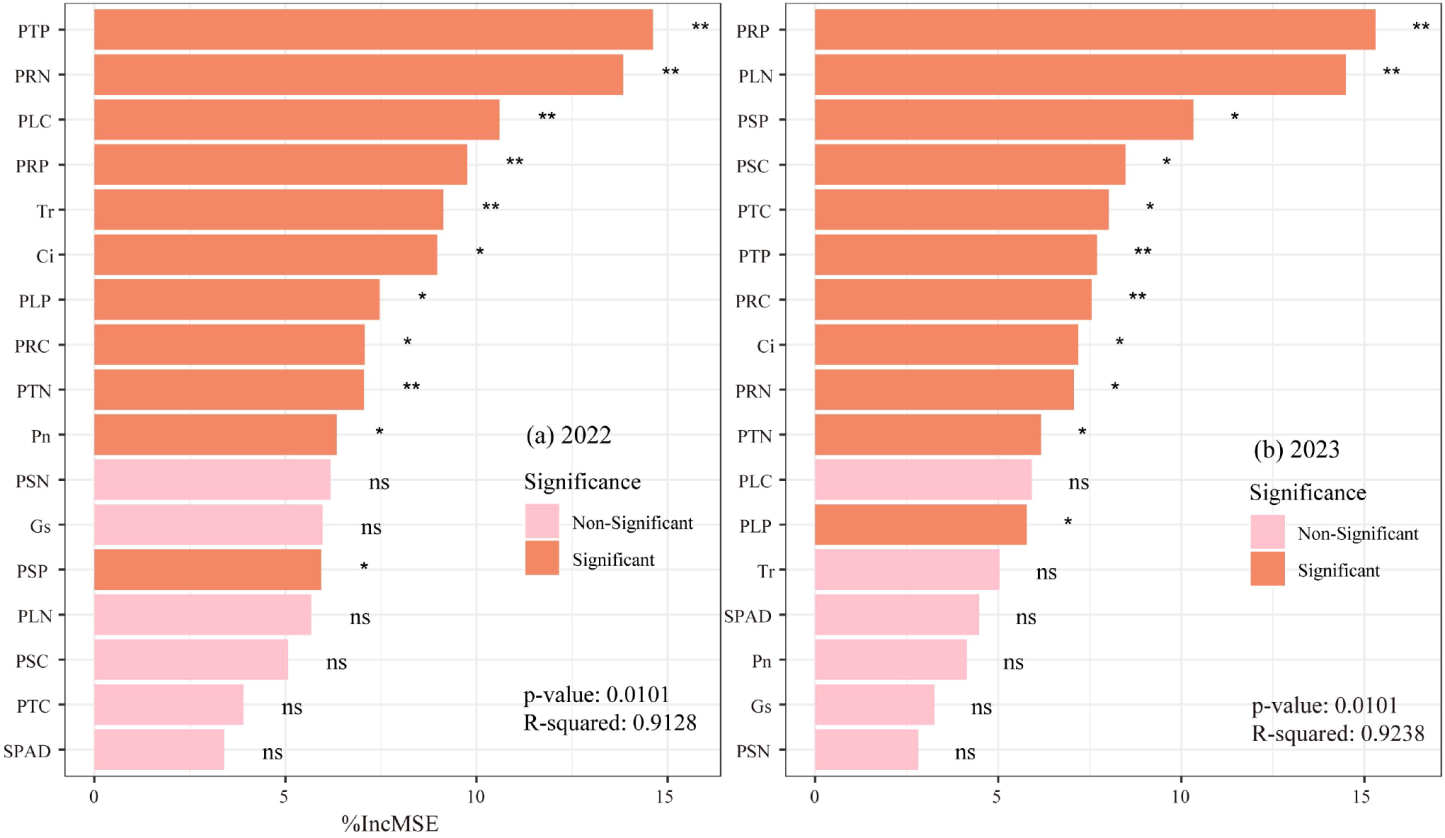
**(a, b)** Relative importance of various indicators on potato yield predicted by random forest model.

Random forest modeling predicted crop yield using SPAD, Tr, Pn, Gs, Ci, PSN, PLN, PRN, PTN, PSP, PLP, PRP, PTP, PSC, PLC, PRC, and PTC (**Figure 13**). The 2022 model (**Figure 13a**) identified PTP, PRN, PLC, PRP, and Tr as key yield determinants, while the 2023 model (**Figure 13b**) highlighted PRP, PLN, PSP, PSC, PTC, PTP, and PRC as major influencing factors.

### 3.7 Evaluation and optimization of water-nitrogen regimes based on binary quadratic regression model and TOPSIS model

**Figure 14:**
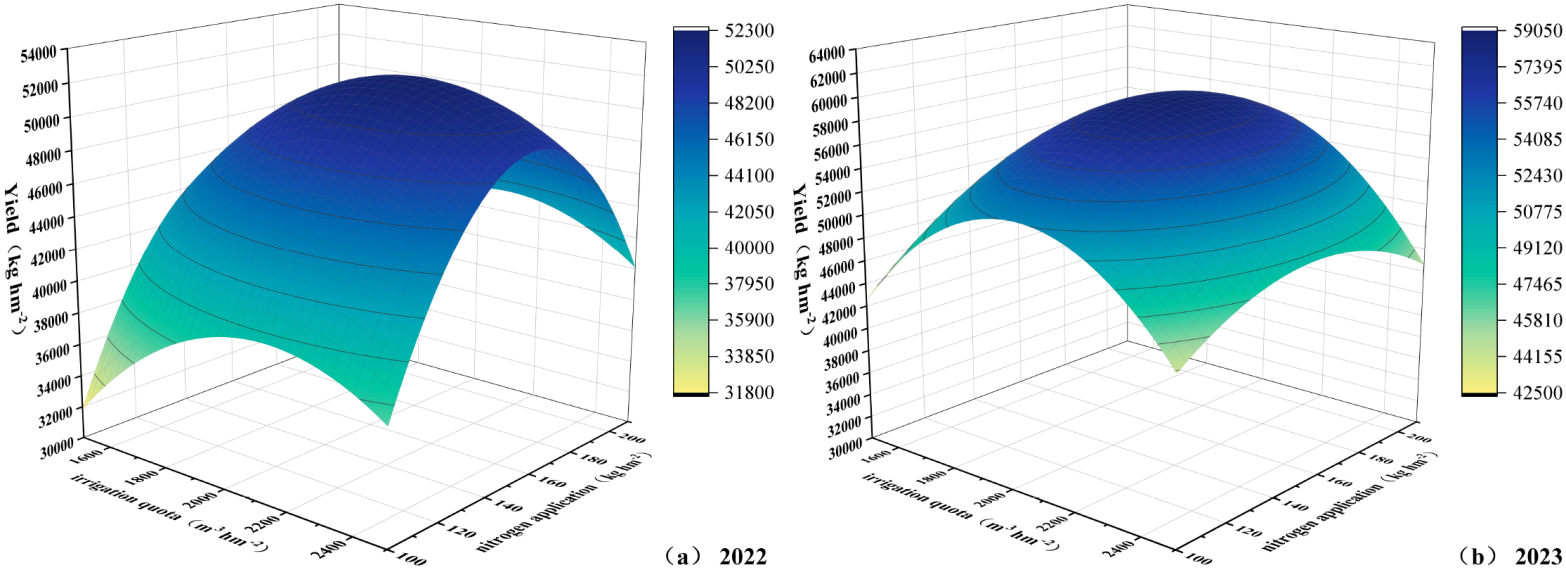
**(a, b)** Relationship between irrigation quota, nitrogen application rate, and yield.

Taking yield (Y) as the target dependent variable, with irrigation quota and nitrogen application rate as dependent variables, and irrigation water amount (W) and pure nitrogen application rate (N) as independent variables, regression analysis was performed on the data to establish a binary quadratic regression equation model (retaining three decimal places). In the 2022 experiment, the model formula for irrigation quota, nitrogen application rate, and yield was Y2022 = -128779.309 + 82.698W + 1174.116N - 0.019W² - 3.569N² - 0.017W·N, with R²=0.9788 and P = 0.0001. In the 2023 experiment, the model formula was Y2023 = -143778.716 + 155.395W + 595.990N - 0.037W² - 1.432N² - 0.059W·N, with R²=0.9650 and P = 0.0001. The R² values were all greater than 0.85, and predicted values showed significant correlation with measured values, indicating that the established models could effectively predict the interactive effects of water and nitrogen on yield. As shown in **Figure 14**, the irrigation amount and nitrogen application rate at maximum yield were 2107.84 m^3^ ha^-1^ and 159.93 kg ha^-1^ in 2022, and 1966.25 m^3^ ha^-1^ and 167.86 kg ha^-1^ in 2023. Comprehensive analysis recommended the W2 and W3 irrigation treatments and N2 nitrogen application treatment.

To further optimize the water-nitrogen regime, the TOPSIS model was employed using 18 indicators including yield, *SPAD*, *Tr*, *Pn*, *Gs*, *Ci*, *PSN*, *PLN*, *PRN*, *PTN*, *PSP*, *PLP*, *PRP*, *PTP*, *PSC*, *PLC*, *PRC*, and *PTC* to select the most efficient irrigation and nitrogen fertilization scheme for potatoes. **Table 8** presents the score ranking table for each treatment. Analysis revealed that the W2N2 treatment achieved the highest total score, while the CK treatment had the lowest score. Under the experimental conditions, the W2N2 treatment is recommended as the optimal water-nitrogen coupling pattern for potato cultivation in the central arid zone of Ningxia.

**Table 8:** Statistical data of TOPSIS evaluation indicators.

Year	Treatments	D^+^	D^-^	Ci*	Rank	Year	Treatments	D^+^	D^-^	Ci*	Rank
2022	W1N1	0.6273	0.1809	0.2238	9	2023	W1N1	0.6501	0.1404	0.1776	9
	W1N2	0.3806	0.4528	0.5433	6		W1N2	0.4383	0.3551	0.4476	7
	W1N3	0.4697	0.3463	0.4243	8		W1N3	0.4837	0.3017	0.3841	8
	W2N1	0.3723	0.4159	0.5277	7		W2N1	0.4055	0.4095	0.5024	5
	W2N2	0.047	0.7527	0.9412	1		W2N2	0.1199	0.7415	0.8608	1
	W2N3	0.2149	0.5842	0.7311	3		W2N3	0.2257	0.5811	0.7202	2
	W3N1	0.3517	0.4662	0.57	5		W3N1	0.4194	0.395	0.485	6
	W3N2	0.1286	0.6574	0.8364	2		W3N2	0.2405	0.5706	0.7035	3
	W3N3	0.2705	0.5234	0.6593	4		W3N3	0.3577	0.4661	0.5658	4
	CK	0.756	0.0348	0.044	10		CK	0.7605	0.0725	0.0871	10

## 4 Discussion

### 4.1 Effects of water-nitrogen interaction on gas exchange parameters in potato leaves

A reasonable irrigation system helps maintain soil moisture balance (Xu et al., 2017). In this study, the W2N2 treatment showed the highest relative chlorophyll content, with an average increase of 2.87% compared to the CK treatment at all growth stages over the two-year experiment. This indicates that appropriate water and nitrogen management can increase the SPAD value (Ma et al., 2023) and maintain a relatively high chlorophyll content. When nitrogen supply is sufficient, the synthesis of chlorophyll in leaves increases, and the SPAD value rises accordingly. Transpiration in potato leaves is a process where water diffuses into the atmosphere in gaseous form. Through transpiration, plants lose over 97% of the water they absorb, highlighting the vital significance of water for plant life activities (Chaves et al., 2016; Griffani et al., 2024). The study found that under water-nitrogen interaction, the W2N2 treatment was significantly higher than other treatments, with an average increase of 228.49% compared to the CK treatment at all growth stages over the two years. This indicates that water-nitrogen interaction can enhance the absorption and transport of water by potato leaves and regulate the transpiration rate of potato leaves. Different water treatments affect the water status of potato plants, thereby influencing the absorption and transport of water by plants as well as the leaf transpiration rate (Zhang et al., 2018). Increased nitrogen application leads to increased biomass accumulation and excessive canopy leaf area, resulting in excessive increases in transpiration and water loss, and ultimately excessive consumption of soil moisture (Wang et al., 2020). The net photosynthetic rate of potato leaves plays a crucial role in plant organic matter accumulation, water regulation, and nutrient distribution, serving as a key physiological indicator of plants (Zahoor et al., 2017). Under water-nitrogen interaction, the W2N2 treatment showed a significantly higher net photosynthetic rate than other treatments, with an average increase of 150.09% compared to the CK treatment across all growth stages over the two years. This indicates that an appropriate supply of water and nitrogen can maintain the normal metabolic activities of leaf cells, ensuring the smooth progress of photosynthesis. Stomatal conductance of potato leaves refers to the degree of stomatal opening, which is a key factor affecting potato photosynthesis, respiration, and transpiration (Damour et al., 2010). Under water-nitrogen interaction, the leaf stomatal conductance of the W2N2 treatment was significantly higher than that of other treatments, with an average increase of 186.49% compared to the CK treatment across all growth stages over the two years. This indicates that when water is sufficient, the stomata of potato leaves usually remain relatively open, facilitating gas exchange. Nitrogen supply can promote the growth and development of potato leaves, increasing the number of stomata and their opening degree; however, either excessive or insufficient nitrogen may have adverse effects on stomatal conductance (Querejeta et al., 2022). The intercellular CO_2_ concentration in potato leaves refers to the concentration of carbon dioxide within the leaf, which is essential for photosynthesis as potatoes absorb CO_2_ to drive this process—directly influencing the rate and efficiency of photosynthesis, and thereby affecting potato growth and development (Shah et al., 2017). Under water-nitrogen interaction, the W2N2 treatment showed a significantly higher intercellular CO_2_ concentration than other treatments, with an average increase of 63.09% compared to the CK treatment across all growth stages over the two years. When water and nitrogen supplies are sufficient, potato leaf stomata remain open, facilitating CO_2_ entry into leaf cells, which in turn. The ratio of photosynthetic rate to transpiration rate provides insight into the water use efficiency (WUE) of potato leaves. This study revealed that WUE reflects the efficiency with which leaves utilize water during photosynthesis: higher WUE indicates stronger growth capacity of potatoes in arid regions. Thus, water-nitrogen interaction can regulate the WUE of potato leaves, thereby promoting potato growth (Wang et al., 2018).

### 4.2 Effects of water-nitrogen interaction on plant nutrient stoichiometry and yield

The content, accumulation, and distribution of carbon (C), nitrogen (N), and phosphorus (P) in different organs are constrained by growth conditions and their own structural characteristics (Sanchez-Bragado et al., 2017). The concentrations and ratios of C, N, and P in plants are important indicators of ecological processes (Liu and Lu, 2024). Nitrogen (N) uptake is regulated by water availability. Water deficiency can limit crop nitrogen response by reducing nitrogen uptake and utilization. The complex and multi-faceted interactions between water supply and crop nitrogen response make it difficult to predict and quantify the impact of water deficiency on crop nitrogen status (Zhao et al., 2024a). This study found that water-nitrogen interaction significantly affects the uptake and distribution of C, N, and P nutrients in potato plants. The level of nitrogen supply directly affects carbon metabolism: appropriate nitrogen application (N2 treatment) enhances photosynthesis by promoting chlorophyll synthesis, thereby improving leaf carbon assimilation capacity. However, excessive nitrogen application (N3 treatment) stimulates excessive growth of aboveground parts, leading to over-distribution of photosynthates to stems and leaves and a reduction in carbon accumulation in tubers. Phosphorus uptake is greatly affected by water conditions. Increasing phosphorus application during the tuber expansion stage can improve nutrient use efficiency, and optimizing the nitrogen-phosphorus ratio can promote plant growth (Rosell et al., 2023). The interactive effect of nitrogen and water is obvious: under drought conditions, high nitrogen levels will exacerbate the imbalance of carbon and nitrogen metabolism, while appropriate irrigation (W2) can alleviate nitrogen stress and promote the transport of nitrogen and phosphorus to tubers. Water-nitrogen interaction significantly affects the accumulation of organic carbon in potato plants by altering the efficiency of photosynthetic carbon assimilation and distribution. Among all water-nitrogen interaction treatments, the W2N2 treatment showed the highest organic carbon accumulation. Reasonable water and nitrogen combination can optimize carbon metabolism pathways, increase the activity of starch synthesis-related enzymes, and improve the carbon conversion efficiency in tubers.

The synergistic effect of water and nitrogen affects the distribution of organic carbon by regulating plant metabolic processes. In 2022, the proportion of organic carbon in stems and roots was relatively high, which may be related to the enhanced supply of water and nitrogen promoting the transport of photosynthates to structural organs (stems and roots). In 2023, however, the increased proportion of organic carbon in leaves and roots reflects the improvement of photosynthetic carbon assimilation efficiency and the enhancement of root carbon storage capacity after water and nitrogen optimization. The characteristic that total nitrogen distribution is dominated by leaves is consistent with the function of leaves as the core organ of nitrogen metabolism. The interaction of water and nitrogen promotes the preferential enrichment of nitrogen in metabolically active leaves by affecting the pathways of nitrate absorption, reduction, and amino acid synthesis (Wang et al., 2022). The lowest proportion of nitrogen in stems is related to their lower nitrogen demand. The dynamic distribution of total phosphorus is closely related to the energy metabolism demand driven by water and nitrogen. The differences in the proportions of phosphorus in roots and stems among different treatments suggest that there is a threshold effect of water status on the radial transport of phosphorus.

The stoichiometry of carbon (C), nitrogen (N), and phosphorus (P) (C:N:P) in leaves, stems, and roots reflects the trade-offs between plant resource acquisition and their growth strategies (Yin et al., 2021). The water-nitrogen interaction drives nutrient allocation and metabolic coordination among organs by regulating the stoichiometric characteristics of C, N, and P (Zheng et al., 2023). The water-nitrogen ratio in the W2N2 treatment can maximize metabolic efficiency and yield formation, providing a theoretical basis for precise water and nitrogen management in potato production. The carbon-nitrogen ratio in stems decreased by 5.25% to 39.96%, indicating that the synergistic effect of water and nitrogen promoted nitrogen enrichment in stems, which may be related to the developmental needs of their vascular tissues and the improved efficiency of water transport. The relatively high carbon-nitrogen ratio in roots reflects the enhanced carbon deposition in roots under water regulation, while the carbon-nitrogen ratio in tubers ranged from 77.07% to 96.79%, suggesting that moderate water restriction combined with high nitrogen supply can optimize the reallocation of carbon to tubers. The increased fluctuation in the leaf carbon-nitrogen ratio indicates a dynamic balance between photosynthetic carbon assimilation and nitrogen metabolism. Among all treatments, the leaf carbon-nitrogen ratio in the W3N3 treatment increased significantly, which may be associated with the promotion of chlorophyll synthesis by high nitrogen levels. The nitrogen-phosphorus ratio in tubers was positively correlated with yield (with a 64.71% yield increase in the W2N2 treatment), further illustrating the contribution of efficient nitrogen and phosphorus utilization to yield formation. The water-nitrogen interaction had the most significant impact on potatoes during the tuber formation stage. This period is a critical turning point for nutrient allocation to tubers, and the synergy of water and nitrogen directly affects the number and initial size of tubers by regulating the transport of photosynthates, the enlargement of stolons, and nutrient accumulation. Adequate water can improve nitrogen use efficiency, and reasonable nitrogen supply can enhance water absorption; an imbalance between the two will hinder tuber development, exerting the most prominent restrictive effect on yield formation (Ma et al., 2023). The nitrogen-phosphorus stoichiometric ratio (N:P) is a key indicator for assessing plant nutrient limitations. For potato leaves, an N:P ratio<14 indicates phosphorus limitation, >16 indicates nitrogen limitation, and a ratio between 14 and 16 indicates nitrogen-phosphorus balance or co-limitation (Güsewell, 2004). Zebarth et al. (2004) reported that potatoes absorb nitrogen rapidly during the seedling stage, and the leaf N:P ratio is often higher than 16, suggesting a high risk of nitrogen limitation. Phosphorus limitation is rare at the seedling stage unless the soil phosphorus level is extremely low. Bélanger et al. (2002) found that an increase in the leaf N:P ratio during the maturity stage is a normal phenomenon, but a sustained ratio higher than 16 may indicate excessive nitrogen fertilization.

In potato planting systems, water and nitrogen supply are crucial factors controlling production levels, especially in arid and semi-arid regions (Badr et al., 2012). Excessive irrigation and nitrogen application can have negative impacts on the yield and quality of potato tubers; overly high levels of irrigation and nitrogen are not conducive to yield formation. The mutual promotion of water and nitrogen on potato yield is consistent with the results of this study (Shrestha et al., 2023). Yield showed a trend of first increasing and then decreasing with the water and nitrogen gradients, revealing that there exists an optimal threshold for water-nitrogen interaction. The W2N2 treatment achieved an average yield increase of 65.08% over the two years. Optimizing the water and nitrogen regime balances the relationship between photosynthesis-stoichiometry synergy and yield formation. The water-nitrogen interaction (W2N2) can simultaneously realize physiological regulation and yield maximization (Wang et al., 2024), providing both theoretical and practical support for precise water and nitrogen management of potatoes in arid regions.

## 5 Conclusions

Water-nitrogen interaction significantly improved the photosynthetic performance of potatoes. Compared with the control (CK), the W2N2 treatment increased leaf transpiration rate (*Tr*), net photosynthetic rate (*Pn*), and stomatal conductance (*Gs*) by an average of 228.49%, 150.09%, and 186.49%, respectively, with photosynthetic indicators peaking during the tuber formation stage. In terms of nutrient content, the W2N2 treatment increased the total nitrogen content in tubers by an average of 121.91% compared with CK, while the total nitrogen in stems, leaves, and roots increased by 55.61%–90.95%. The organic carbon content was the highest in tubers. The water-nitrogen interaction had a significant effect on the leaf transpiration rate of potatoes, with a 63.09% increase in intercellular CO_2_ concentration (*Ci*) during the tuber formation stage. The W2N2 treatment (irrigation quota: 2107.84–1966.25 m^3^ ha^-1^; nitrogen application rate: 159.93–167.86 kg ha^-1^) achieved the optimal synergy among photosynthesis, carbon-nitrogen metabolism, and yield. Compared with CK, the yield increased by 64.71% in 2022 and 65.84% in 2023.

## Data Availability

The original contributions presented in the study are included in the article/supplementary material. Further inquiries can be directed to the corresponding authors.
